# Genome-wide analysis of the MYB transcription factor superfamily in soybean

**DOI:** 10.1186/1471-2229-12-106

**Published:** 2012-07-09

**Authors:** Hai Du, Si-Si Yang, Zhe Liang, Bo-Run Feng, Lei Liu, Yu-Bi Huang, Yi-Xiong Tang

**Affiliations:** 1 Maize Research Institute, Sichuan Agricultural University, Chengdu Sichuan, China; 2 Key Laboratory of Biology and Genetic Improvement of Maize in Southwest Region, Maize Research Institute of Sichuan Agricultural University, Ministry of Agriculture, Chengdu Sichuan, China; 3 Biotechnology Research Institute, Chinese Academy of Agricultural Sciences, Beijing, China; 4 Department of Plant and Environmental Sciences, Norwegian University of Life Sciences, PO Box 5003N-1432, Norway

## Abstract

**Background:**

The MYB superfamily constitutes one of the most abundant groups of transcription factors described in plants. Nevertheless, their functions appear to be highly diverse and remain rather unclear. To date, no genome-wide characterization of this gene family has been conducted in a legume species. Here we report the first genome-wide analysis of the whole MYB superfamily in a legume species, soybean (*Glycine max*), including the gene structures, phylogeny, chromosome locations, conserved motifs, and expression patterns, as well as a comparative genomic analysis with *Arabidopsis*.

**Results:**

A total of 244 R2R3-MYB genes were identified and further classified into 48 subfamilies based on a phylogenetic comparative analysis with their putative orthologs, showed both gene loss and duplication events. The phylogenetic analysis showed that most characterized MYB genes with similar functions are clustered in the same subfamily, together with the identification of orthologs by synteny analysis, functional conservation among subgroups of MYB genes was strongly indicated. The phylogenetic relationships of each subgroup of MYB genes were well supported by the highly conserved intron/exon structures and motifs outside the MYB domain. Synonymous nucleotide substitution (*d*_N_/*d*_S_) analysis showed that the soybean MYB DNA-binding domain is under strong negative selection. The chromosome distribution pattern strongly indicated that genome-wide segmental and tandem duplication contribute to the expansion of soybean MYB genes. In addition, we found that ~ 4% of soybean R2R3-MYB genes had undergone alternative splicing events, producing a variety of transcripts from a single gene, which illustrated the extremely high complexity of transcriptome regulation. Comparative expression profile analysis of R2R3-MYB genes in soybean and *Arabidopsis* revealed that MYB genes play conserved and various roles in plants, which is indicative of a divergence in function.

**Conclusions:**

In this study we identified the largest MYB gene family in plants known to date. Our findings indicate that members of this large gene family may be involved in different plant biological processes, some of which may be potentially involved in legume-specific nodulation. Our comparative genomics analysis provides a solid foundation for future functional dissection of this family gene.

## Background

Transcription factors are usually composed of at least four discrete domains: a DNA-binding domain, a nuclear-localization signal, a transcription-activation domain, and an oligomerization site. These domains operate together to regulate many physiological and biochemical processes, and to activate and/or repress transcription in response to endogenous and exogenous stimuli [1,2]. In addition, transcription factors are usually encoded by multigene families, thereby multiplying the number and complexity of possible transcriptional regulatory roles [1].

MYB transcription factors are widely distributed in all eukaryotic organisms, and constitute one of the largest transcription factor families in the plant kingdom. MYB proteins are defined by a highly conserved MYB DNA-binding domain at the N-terminus [3]. The MYB domain is highly conserved among animals, yeasts, and plants, and typically consists of 1–4 imperfect repeats (R0, R1, R2, and R3). Each repeat contains approximately 50–53 amino acids and encodes three α-helices, with the second and third helices forming a helix–turn–helix (HTH) structure. When bound to DNA, the HTH structure intercalates in the major groove [3,4]. Moreover, it contains three regularly spaced tryptophan residues, which form a cluster in a hydrophobic core of each repeat, and stabilize the structure of the DNA-binding domain [5]. By contrast, the C-terminus is the activation domain and varies considerably between MYB proteins, which leads to the wide range of regulatory roles of the MYB gene family [4,6,7].

The first identified MYB gene was the v-*myb* gene of the avian myeloblastosis virus [8]. Subsequently, three v-*myb*-related genes (c-*myb*, A-*myb*, and B-*myb*) were identified in diverse vertebrates, insects, fungi, and slime molds [3,9-11]. In general, the animal MYB DNA-binding domain consists of three tandem repeats of the MYB motif (designated R1, R2, and R3), which are referred to as 3R-MYB proteins. The first plant MYB gene identified was C1, which was isolated from *Zea mays* and encodes a c-*myb*-like transcription factor involved in anthocyanin biosynthesis [12]. An increasing number of plant R2R3-MYB superfamily members have been identified subsequently and characterized in numerous plants. The diverse functions of these genes in plant-specific processes include secondary metabolism [13-16], hormone signal transduction [17,18], environmental stresses [19-21], cell shape, and organ development [22-25]. In addition, although plants may also contain 3R-MYB genes, the major MYB transcription factors are the R2R3-MYB and/or R1-MYB types [26,27]. For example, the *Arabidopsis thaliana* genome contains only five 3R-MYB genes, compared with up to 190 R2R3-MYB and MYB-related genes [4,27,28]. Meanwhile, the *Populus* genome contains five 3R-MYB genes and 192 R2R3-MYB genes [29].

Presently, most of our knowledge of plant MYB genes was obtained from studies of the major genetic model plant, *Arabidopsis*, based on its well-annotated genome sequence. However, this genus lacks certain agricultural traits, such as the ability to form nitrogen-fixing symbiotic associations with rhizobia, and soil nutrient-scavenging symbiotic associations with mycorrhizal fungi. Thus, although it constitutes a useful model organism for gene functional studies, it has limited potential agricultural applications. By contrast, legumes are able to establish beneficial nitrogen-fixing and mycorrhizal symbiotic associations and, as a result, have been mainstays for sustainable agriculture for thousands of years. However, to date, no genome-wide characterization of the MYB family has been conducted in a legume species. Consequently, as discussed below, the functions of only a few leguminous MYB genes (MYBs) are known, mainly those related to abiotic stresses, flavonoid metabolism, and nodulation. Furthermore, the genome-wide distribution and phylogenetic relationship of soybean MYB genes (GmMYBs) with other plant MYBs remains poorly understood. Fortunately, the completed genome sequence of soybean provides a valuable resource for genomic analyses of this gene family. Thus, there is an urgent need to characterize the roles of MYBs in soybean and to achieve complete identification and classification of these genes.

Given the potential roles of MYB proteins in regulation of gene expression, secondary metabolism, and responses to environmental stresses, and that soybean is the first legume for which the complete genome has been sequenced, it is of interest to determine how many MYB genes exist in this species, as well as the characteristics of gene structures, phylogenetic relationships, chromosome locations, conserved motifs and expression patterns. Moreover, it is important to unravel the functionality of new and as yet uncharacterized soybean MYBs, especially those that might be involved in seed development and legume-specific nodulation. Within this framework, in the present study we identified the entire complement of MYBs in soybean and conducted a genome-wide comparative analysis of MYBs between soybean and *Arabidopsis*. We present a comprehensive classification, together with structural, expressional, and functional analyses, of the soybean MYB gene family. We identified a total of 252 MYBs, including 244 R2R3-MYB (2R-MYB) genes, six R1R2R3-MYB (3R-MYB) genes, and two R0R1R2R3-MYB (4R-MYB) genes, most of which have not been functionally characterized previously. On the basis of a phylogenetic analysis, we divided the MYBs in *Arabidopsis* and soybean into 48 subfamilies, which allowed identification of shared and unique subfamilies, and examination of functional relationships among subgroups of MYBs. We also estimated the number of MYBs in the most recent common ancestor of soybean and *Arabidopsis*. It is worth noting that the reliability of the subfamilies defined on the basis of the phylogenetic analysis was supported by additional criteria, such as the presence and position of introns, gene structure, and presence of common protein motifs outside the MYB domain. Chromosomal distribution analysis revealed that some of the duplication events are likely to have contributed to the expansion of this gene family. The particular clusters of paralogous and orthologous genes identified within each subfamily showed ancestral duplication and/or gene loss events. Furthermore, the expression profiles of different organs, tissues, and/or developmental stages were previously revealed by analyses of soybean whole-genome RNA-seq [30]. Comparative analysis of the expression profiles of MYBs in soybean and *Arabidopsis* showed that AtMYBs and GmMYBs exhibit various expression patterns, and that MYBs in the same phylogenetic clades and subclades have conserved expression patterns. This finding suggests the existence of functional diversification and conservation between GmMYBs and their close homologs. Moreover, there are species-specific subfamilies, some of which are specifically expressed during nodulation. Our findings constitute the first step towards further investigations into the biological and molecular functions of MYB transcription factors in soybean.

## Results and discussion

### Identification and classification of the soybean MYB gene family

With the aim of defining the soybean MYB gene family, we searched the entire soybean genome sequence for genes that encode proteins containing the MYB DNA-binding domain. BLASTP searches were performed using consensus-typical R2R3-MYB DNA-binding domain sequences as queries. We identified approximately 700 sequences that contain MYB or MYB-like repeats; these represented approximately 1.7% of the 46,430 predicted soybean protein-coding loci, and 14% of the 5671 putative soybean transcription factor genes [31]. Initially, we removed from the data set the redundant sequences on the basis of their identification numbers and chromosome locations.

In order to verify the reliability of our results, we performed PROSITE profile (PS50090) and simple modular architecture research tool (SMART) analysis to identify all of the putative MYB protein sequences in the soybean genome. We excluded from further analysis 26 putative MYB protein sequences that contained typical R2R3-MYB domains but lacked partial sequences in the N-terminal region ( Additional file 1). We also excluded Glyma09g37340, which contained a predicted MYB motif and intact open reading frame (OFR) with low confidence value. The putative novel MYB sequences, combined with the previous estimates of MYB families, finally yielded from the soybean genome a primary data set of 244 typical R2R3-MYB proteins (2R-MYB), six R1R2R3-MYB proteins (3R-MYB), and two 4R-like MYB proteins (4R-MYB) ( Additional file 1). It was defined that the MYB superfamily is the most abundant transcription factor family in plants, with 126 2R-MYB genes in *Arabidopsis*[4], 109 in rice[27,32], 118 in grape[29,33], and 192 in Populus[29]. In this study, the remaining 252 sequences (more than 4% of the 5671 predicted soybean transcription factor genes) represented an updated classification of the MYB family in soybean, and constituted one of the largest known plant MYB transcription factor gene families. The resulting sequences were named according to the generic system, and the correspondence of the sequence names with gene and protein identifiers from the corresponding genome browsers ( Additional file 1). The nomenclature system for GmMYBs used in the present study was provisionally applied to distinguish each of the MYB genes.

### Multiple sequence alignment and sequence features of the MYB domains

**Figure 1:**
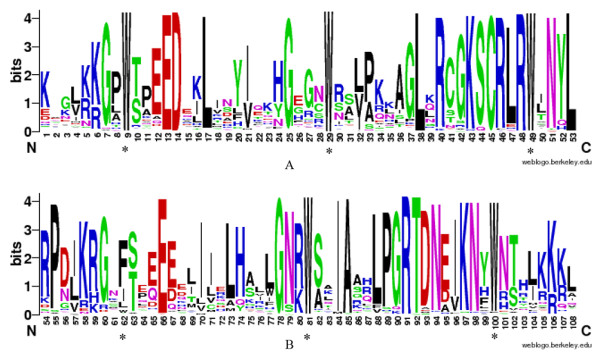
** R2 and R3 MYB repeats are highly conserved across all R2R3-MYB proteins in the soybean genome.** The sequence logos of the R2 (**a**) and R3 (**b**) MYB repeats are based on full-length alignments of all soybean R2R3-MYB domains. Multiple alignment analysis of 244 soybean-typical R2R3-MYB domains was performed with ClustalW (for full representation of the alignment, see Additional file 2). The bit score indicates the information content for each position in the sequence. Asterisks indicate the conserved tryptophan residues (Trp) in the MYB domain.

To investigate the homologous domain sequence features, and the frequency of the most prevalent amino acids at each position within each repeat of the soybean R2R3-MYB domain, we performed multiple alignment analysis using the 244 homologous domain amino acid sequences of R2 and R3 repeats (Figure 1).

In general, the basic regions of MYB domains had 108 basic residues (including the linker region), with rare deletions or insertions (approximately 6%; Additional file 2). By contrast, the regions outside the DNA-binding domain were divergent, in terms of length and also amino acid composition. Figure 1 shows the distribution of amino acid residues at the corresponding positions of the R2 and R3 MYB repeats. Generally, the distribution of conserved amino acids among the MYB domains of soybean were very similar to those of *Arabidopsis*, as expected from the evolutionary distances for MYBs among plants. Consistent with earlier reports, the R2 and R3 MYB repeats of the soybean R2R3-MYB family contained characteristic amino acids, including a series of evenly distributed and highly conserved tryptophan (Trp) residues, which are known to play a key role in sequence-specific DNA binding [4,29,32]. In addition to the highly conserved Trp amino acid residues, we observed alternative highly conserved residues in more than 90% of the soybean R2R3-MYB domains. These included Gly-7, Glu-13, Asp-14, Leu-17, Gly-25, Leu-38, Arg-40, Lys-43, Cys-45, Arg-46, Arg-48, Asn-51, and Leu-53 in the R2 repeat (Figure 1a) and Pro-55, Glu-66, Gly-78, Ile-84, Ala-85, Leu-88, Pro-89, Gly-90, Arg-91, Thr-92, Asp-93, Asn-94, Lys-97, and Asn-98 in the R3 repeat (Figure 1b). As with its counterparts in other plant species, the first conserved Trp residue in the R3 repeat was generally replaced by F (Figure 1b). As shown in Figure 1, the major conserved residues in the MYB domain were mainly distributed between the second and third conserved Trp residues in each repeat. Thus, the first part of each repeat in the MYB domain was apparently less conserved, mainly because the third helix of the HTH domain in each repeat was the most conserved region among the 244 GmMYBs.

**Figure 2:**
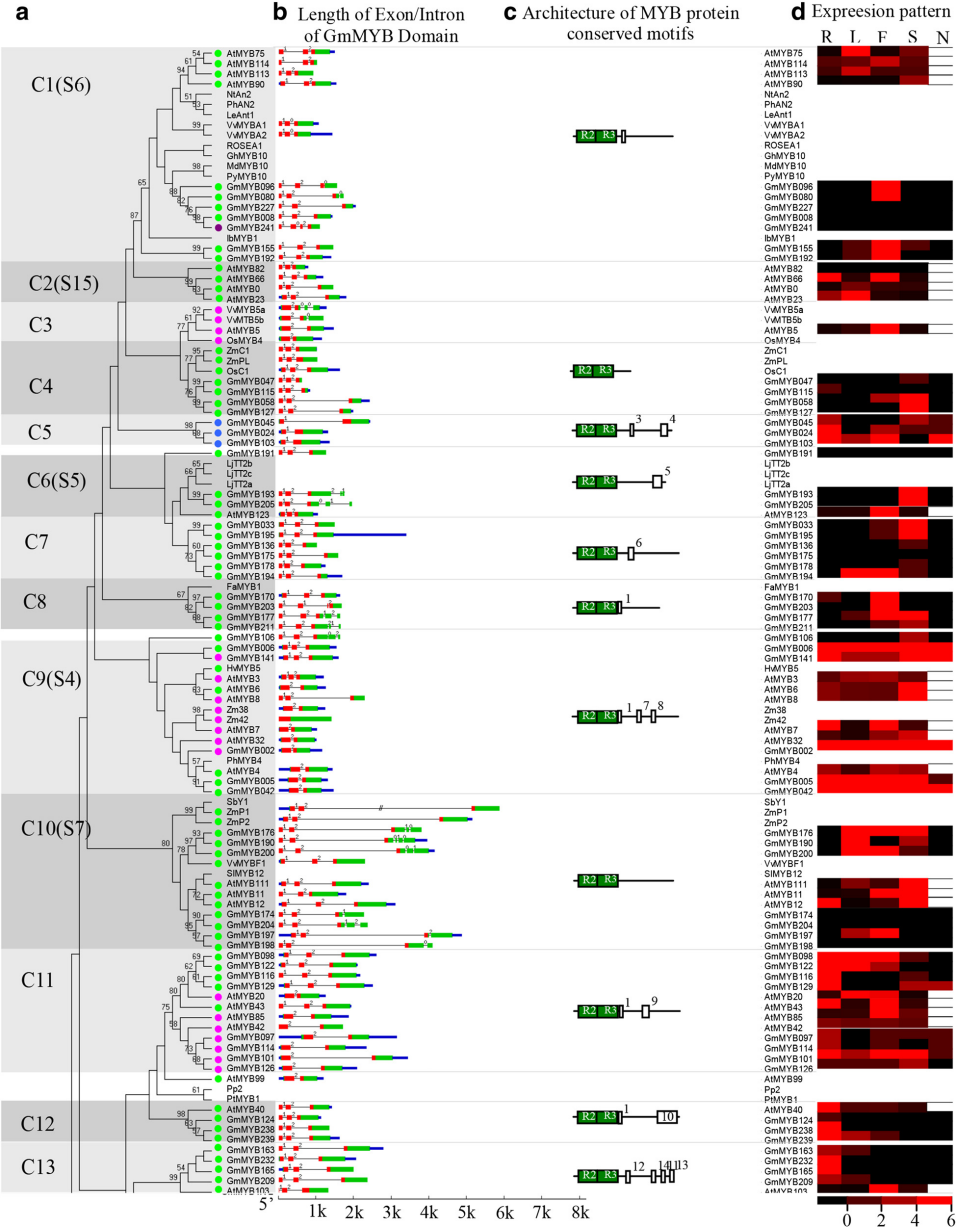
** Phylogenetic relationships and subgroup designations in MYB proteins from soybean (Gm),*Arabidopsis*(At) and other plants.** (**a**) Neighbor-joining tree representing relationships among 252 MYB proteins from soybean and 132 MYB proteins from *Arabidopsis*, including five 3R-MYB proteins from *Arabidopsis* and six 3R-MYB proteins from soybean. The proteins are clustered into 47 subgroups, which are designated with a subgroup number (e.g., C1) and marked with different alternating tones of a gray background to facilitate subfamily identification with high predictive value. The numbers beside the branches represent bootstrap support values (>50%) from 1000 replications. Sixteen proteins did not fit well into clusters. Colored circles indicate the corresponding intron distribution patterns, as shown in Figure 3. (**b**) Structure of MYB genes in soybean and *Arabidopsis*. Exon(s) are indicated by green boxes, MYB domain(s) by red boxes, untranslated region(s) by blue boxes, and spaces between the colored boxes correspond to introns. The sizes of exons and introns can be estimated using the horizontal scale bar. (**c**) Architecture of conserved protein motifs in 47 subfamilies. The motifs on the right were detected using MEME and are graphically represented as white boxes drawn to scale for a representative plant MYB protein of each subfamily. (**d**) Expression patterns of MYB genes in soybean and *Arabidopsis* in different organs. R, root; L, leaf; F, flower; S, seed; N, legume-specific nodulation. In this expression pattern analysis, the highest values among the expression values of the four organs published in the AtGenExpress and SoySeq databases were selected.

Sequence similarity among the 244 GmMYBs at the cDNA level showed a high degree of sequence divergence ranging from 5.50% to 99% in the open reading frame (ORF). The highest similarity in the ORF was observed between GmMYB100, GmMYB243, and GmMYB244, whereas the lowest similarity was observed between GmMYB029 and GmMYB156. Most of the genes had very short 5′-untranslated regions (UTR) (Figure 2b and Additional file 3), and showed the lowest similarity in that region, which indicated that the coding region was more conserved than the 5′-UTR sequence. The 3′-UTR sequences were similarly less conserved among the GmMYBs. In addition, we observed that some 5′- and 3′-UTR sequences of GmMYBs had introns. Interestingly, these genes were mainly clustered in the same subfamily (C40; Additional file 3). The predicted proteins encoded by the 244 R2R3-MYBs shared approximately 25–100% identity. As with MYB proteins in other plants, the MYB proteins of soybean were highly conserved in the DNA-binding domain. The overall similarity in the MYB domain of the 244 GmMYB proteins appeared to be relatively high. About 11 pairs of GmMYBs proteins were almost identical in the MYB domains, whereas the similarity of more than 72 pairs of GmMYBs exceeded 97% (a difference was observed in fewer than three of the 108 residues in the R2R3 region). By contrast, there was little sequence similarity downstream of the MYB domain.

As an indicator of the selective pressures that act on these genes, we calculated ratios of nonsynonymous vs synonymous nucleotide substitutions (*d*_N_/*d*_S_) in the soybean MYB domain. By globally fitting an evolutionary model, a *d*_N_/*d*_S_ estimate of 0.1567 was obtained, which showed that the soybean MYB domain is under strong negative selection (neutral selection corresponds to a ratio of 1). At the individual codon level, we found 99 residues were under negative selection in MYB genes without a single positively selected residue.

### Alternative splicing of the MYB gene

Alternative splicing (AS) is the mechanism by which a common precursor mRNA produces different mRNA variants, by extending, shortening, skipping, or including exon sequences, or retaining intron sequences. This allows production of many gene products with enriched functions from a single coding sequence. Recently, it was proposed to be a mechanism by which higher-order diversity is generated, thereby leading to a diverse population of RNAs [34,35]. Various gene isoforms generated by AS may have specific roles in particular cell compartments, tissues, and stages of development.

About 27% of 18,933 transcription units in rice genomes contain two or more transcripts because of AS [36]. In the present study, AS of MYB genes were detected. We found that 10 of 244 R2R3-MYB genes in soybean contained two to five alternative structures that indicated they had undergone AS, thus producing a variety of transcripts from a single gene ( Additional file 1). Among these genes, five underwent multiple promoters events, four competing 3′ splice sites events, and one a multiple poly(A) sites event. Similarly, up to 18 R2R3-MYB genes in *Arabidopsis* undergo similar AS events, including five multiple promoters events, five competing 5′ splice sites events, four competing 3′ splice sites events, one multiple poly(A) sites event, and one retained intron event. The remaining two AS events occurred in the 5′-UTR region without affecting the coding frame, which indicated they create a variety of UTRs that may play a key role in gene regulation. Our results showed that multiple promoters events and competing 3′ and/or 5′ splice sites events are likely to be the primary type of AS events in R2R3-MYB genes, which is in contrast to human and yeast AS events [37,38]. Our analysis also showed that the average size of exon modification of competing 5′ and 3′ splice sites was significantly short (about 31 bp). More interestingly, in soybean the four competing 3′ splice sites events were all located at the same site in the second helix in the R3 repeat, which indicated that there may be a conserved mechanism.

In general, these AS events resulted in a variety of sequence insertions and/or deletions in the corresponding ORFs. For instance, a 33 bp alternative 3′ splice site in GmMYB028 allowed the lengthening or shortening of 11 amino acids in the R3 repeat. Similarly, a 21 bp alternative 3′ splice site in GmMYB140 resulted in a deletion of seven amino acids in the R3 repeat. Interestingly, we observed that some of the AS events changed the type of MYB protein. For example, a 189 bp alternative promoter site of GmMYB082 resulted in a frame shift, which changed the R2R3-MYB into a signal-repeat MYB type. Similarly, a 237 bp alternative promoter site in GmMYB152 that transposed the 5′ exons changed the typical R2R3-MYB gene into a signal-repeat MYB gene. In contrast, GmMYB237 exhibited three alternative types of splicing by alternative promoters and alternative poly(A) sites. The first transcript resulted in a deletion of more than 2000 bp at the 5′ terminus and a long insertion at the 3′ terminus; the second resulted in a long deletion at the 3′ terminus; and the third (3R-MYB protein) resulted in a deletion of the R1 and R3 repeats in the 3R-MYB protein. In *Arabidopsis*, 16 of the 18 AS events undergo similar AS events (data not shown), which further highlighted the relevance of AS to the MYB gene family.

There are currently two models for the evolution of the MYB DNA-binding domain: the ‘gain’ and the ‘lose’ models. The ‘gain’ model proposes that the R1R2R3-MYBs are generated by successive intragenic duplications or triplications in primitive eukaryotes [3,39]. The duplication of the MYB domain to yield multiple-repeat MYB proteins, followed by expansion of MYB proteins through duplication of entire genes, results in the abundance of R2R3-MYB and R1R2R3-MYB in higher plants and animals, respectively [40]. The ‘lose’ model suggests that plant MYB ancestors may have had three MYB repeats, and that the R2R3-MYB-related proteins arose following the loss of sequences encoding R1 in the ancestral three-repeat MYB gene [26,41]. Each of the two models has advantages and disadvantages for explanation of the origin of the MYB transcription factor family. Thus, the evolution of this gene family requires further investigation. Our present findings indicate that some MYB genes can produce different variants by AS, and that some of these variants involve a change from 2R-MYB to 1R-MYB, or from 3R-MYB to 2R-MYB. Thus, it appears that AS events provide new insights into the evolution of MYB proteins.

### Phylogenetic analysis of the MYB family genes

To evaluate the evolutionary relationships within the MYB gene family, we performed a combined phylogenetic analysis of *Arabidopsis* and soybean R2R3-MYB proteins to obtain a neighbor-joining (NJ) tree (Figure 2a and Additional file 3). The large number of taxa and relatively small number of informative characters resulted in low bootstrap support values for internal nodes; by contrast, the outer nodes received higher bootstrap support (data not shown). Thus, we sought additional evidence to support the reliability of our subfamily designations by using maximum parsimony (MP) analysis to generate a MP tree ( Additional file 4). The tree topologies derived from the NJ and MP analyses were essentially identical, which indicated that the two methods were in strong agreement. We found only three sequences (GmMYB078, GmMYB108, and GmMYB143) that were not members of any of the identified subfamilies or showed ambiguous placements between the different phylogenetic trees (Figure 2a and Additional file 3).

The observed sequence similarity and phylogenetic tree topology allowed us to classify the MYB genes of soybean, *Arabidopsis*, and other plants into 47 subfamilies, which ranged in size from two to 28 MYB genes (Figure 2a and Additional file 3). In our subfamily classification of the MYB genes, we also took into account the results of Stracke et al. (2001) and Dubos et al. (2010) for AtMYBs (Figure 2a and Additional file 3). The validity of our phylogenetic reconstruction is confirmed by the fact that it shows the same subgroups as those observed in previously constructed phylogenetic trees [4,33,42]. In previous studies, 90 of the 126 AtMYBs were grouped into 25 subfamilies. However, the remaining 36 genes were not included in the phylogenetic analysis because of low statistical support. To compare our results with these previous studies, we labeled the clades previously defined in the tree shown in Figure 2 and Additional file 3. Most of the large subfamilies (i.e., C1, C9, and C38) were supported by the previous studies and received strong bootstrap support. However, some small subfamilies (i.e., C11, C15, and C35; Figure 2a and Additional file 3) were not retrieved in the NJ trees reported by Stracke et al. (2001) and Dubos et al. (2010).

In comparisons of the multigene families, the MYBs from the same lineage tended to be clustered together in the phylogenetic tree, which suggested that they were duplicated after the divergence of the lineages. Moreover, soybean and *Arabidopsis* R2R3-MYB proteins were not equally represented within given clades (Figure 2a and Additional file 3). It was common to find that two or more GmMYBs were putative orthologs of a single gene in *Arabidopsis*, which indicated that MYBs in soybean experienced duplications after the divergence of soybean and *Arabidopsis*. For example, the phylogeny for subfamily C22 included only one AtMYB and six GmMYBs. By contrast, four AtMYBs and two GmMYBs were included in subfamily C19. Remarkably, some homologs were clustered by species within a clade (Figure 2a and Additional file 3), which showed ancestral duplication and gene loss events. For example, in subfamily C24, no GmMYBs were grouped within the *Arabidopsis* ‘glucosinolate’ clade [43-45], members of which are predominantly present in Cruciferae species. This suggested the existence of species-specific MYBs that were either lost in *Arabidopsis*, or acquired in the soybean lineages after divergence from the most recent common ancestor. The anatomical and physiological differences between soybean and *Arabidopsis* indicate that some clades may have been differentially expanded when comparing these two R2R3-MYB subfamilies. Within and outside some of these functional clades, soybean gene expansions were observed as gene pairs or clusters. This might reflect the loss of a clade member or misannotation of the soybean genome. In this situation, knowledge of the gene functions of certain members will facilitate confirmation of paralogous and orthologous relationships. As the functions of most GmMYBs have yet to be characterized, it will be useful to reveal the orthologs within each clade.

However, it is difficult to infer orthologs merely from tree topologies. In general, the gene functions of a clade appear highly but not absolutely conserved across plant species. Therefore, it is useful to accurate identify the true orthologs between plant species, based on syntenic relationship. To support the putative orthology of GmMYBs and AtMYBs, we examined the syntenic relationship based on gene content, order, and orientation between the genomes of soybean and *Arabidopsis*. Despite chromosomal rearrangements (e.g., inversion of gene orientation), gene content was similar between the different chromosome regions analyzed, which supported the deduction that these genes are more likely to be orthologous. The results demonstrated that extensive soybean regions exhibit synteny with *Arabidopsis* genome, and as much as 1610 syntenic blocks between soybean and *Arabidopsis* genome were identified in this study. Moreover, up to 100 high confidence syntenic blocks include at least one pair of MYB orthologous genes in these two species, accounting for about 36% of the GmMYBs, such as GmMYB232/AtMYB103, GmMYB138/AtMYB124, and GmMYB214/AtMYB61 (Table 1, and Additional file 5). It is common to find that several GmMYBs located on different chromosomes are orthologs of a same AtMYB gene, which strongly supported the genome duplication event in soybean genome. By contrast, the observation that duplicated AtMYBs are orthologs of a single GmMYB gene suggested that the AtMYBs also experienced duplications after the divergence from the common ancestor.

**Table 1:** **One-to-one orthologous relationships between*Arabidopsis*and soybean**

**Synteny Block**	**AtMYB gene**	**Chr**	**GmMYB gene**	**Chr**	**Synteny Block**	**AtMYB gene**	**Chr**	**GmMYB gene**	**Chr**
1	AtMYB103	1	GmMYB232	1	52	AtMYB11	3	GmMYB190	3
2	AtMYB124	1	GmMYB138	1	53	AtMYB77	3	GmMYB011	4
3	AtMYB61	1	GmMYB214	10	53	AtMYB84	3	GmMYB161	4
4	AtMYB50	1	GmMYB214	10	54	AtMYB57	3	GmMYB027	5
5	AtMYB20	1	GmMYB116	11	55	AtMYB94	3	GmMYB207	5
6	AtMYB103	1	GmMYB165	11	56	AtMYB125	3	GmMYB143	7
7	AtMYB112	1	GmMYB014	17	57	AtMYB26	3	GmMYB102	7
8	AtMYB31	1	GmMYB153	17	58	AtMYB121	3	GmMYB108	8
9	AtMYB124	1	GmMYB091	18	59	AtMYB67	3	GmMYB065	8
10	AtMYB20	1	GmMYB129	18	60	AtMYB26	3	GmMYB107	8
11	AtMYB61	1	GmMYB094	19	61	AtMYB85	4	GmMYB097	1
12	AtMYB20	1	GmMYB098	2	62	AtMYB73	4	GmMYB032	1
13	AtMYB124	1	GmMYB051	2	63	AtMYB42	4	GmMYB097	1
14	AtMYB61	1	GmMYB039	3	64	AtMYB18	4	GmMYB148	10
15	AtMYB31	1	GmMYB172	4	65	AtMYB55	4	GmMYB213	10
16	AtMYB103	1	GmMYB209	5	66	AtMYB42	4	GmMYB114	11
17	AtMYB31	1	GmMYB173	6	67	AtMYB85	4	GmMYB114	11
18	AtMYB103	1	GmMYB163	8	68	AtMYB41	4	GmMYB242	13
19	AtMYB124	1	GmMYB067	8	69	AtMYB85	4	GmMYB126	17
20	AtMYB54	1	GmMYB068	9	70	AtMYB55	4	GmMYB009	19
21	AtMYB88	2	GmMYB138	1	71	AtMYB55	4	GmMYB184	2
22	AtMYB70	2	GmMYB032	1	72	AtMYB55	4	GmMYB185	3
23	AtMYB70	2	GmMYB003	11	73	AtMYB85	4	GmMYB101	5
24	AtMYB104	2	GmMYB075	12	74	AtMYB102	4	GmMYB210	7
25	AtMYB101	2	GmMYB075	12	75	AtMYB74	4	GmMYB210	7
26	AtMYB104	2	GmMYB181	13	76	AtMYB97	4	GmMYB109	8
27	AtMYB25	2	GmMYB081	14	77	AtMYB18	4	GmMYB145	9
28	AtMYB38	2	GmMYB018	19	78	AtMYB44	5	GmMYB032	1
29	AtMYB14	2	GmMYB132	19	79	AtMYB86	5	GmMYB214	10
30	AtMYB12	2	GmMYB200	2	80	AtMYB19	5	GmMYB148	10
31	AtMYB2	2	GmMYB099	3	81	AtMYB59	5	GmMYB074	11
32	AtMYB91	2	GmMYB240	7	82	AtMYB49	5	GmMYB117	12
33	AtMYB77	3	GmMYB032	1	83	AtMYB37	5	GmMYB217	12
34	AtMYB1	3	GmMYB049	1	84	AtMYB49	5	GmMYB079	13
35	AtMYB27	3	GmMYB146	10	85	AtMYB59	5	GmMYB037	13
36	AtMYB45	3	GmMYB148	10	86	AtMYB119	5	GmMYB235	14
37	AtMYB48	3	GmMYB074	11	87	AtMYB78	5	GmMYB028	15
38	AtMYB30	3	GmMYB004	13	88	AtMYB59	5	GmMYB082	15
38	AtMYB35	3	GmMYB119	13	89	AtMYB40	5	GmMYB124	16
39	AtMYB27	3	GmMYB120	13	90	AtMYB24	5	GmMYB125	16
40	AtMYB109	3	GmMYB081	14	91	AtMYB36	5	GmMYB220	17
41	AtMYB77	3	GmMYB019	14	92	AtMYB78	5	GmMYB014	17
41	AtMYB84	3	GmMYB219	14	92	AtMYB111	5	GmMYB204	17
42	AtMYB108	3	GmMYB028	15	93	AtMYB80	5	GmMYB089	18
43	AtMYB67	3	GmMYB085	15	94	AtMYB24	5	GmMYB026	19
44	AtMYB21	3	GmMYB125	16	95	AtMYB40	5	GmMYB238	19
45	AtMYB57	3	GmMYB125	16	96	AtMYB86	5	GmMYB023	20
46	AtMYB94	3	GmMYB153	17	97	AtMYB96	5	GmMYB172	4
47	AtMYB30	3	GmMYB007	18	98	AtMYB78	5	GmMYB105	7
48	AtMYB21	3	GmMYB130	19	98	AtMYB111	5	GmMYB174	7
48	AtMYB35	3	GmMYB131	19	99	AtMYB80	5	GmMYB144	8
49	AtMYB30	3	GmMYB046	19	100	AtMYB78	5	GmMYB110	9
50	AtMYB11	3	GmMYB176	19	100	AtMYB111	5	GmMYB197	9
51	AtMYB45	3	GmMYB025	20					

### Definition of intron/exon structures within the MYB domains

**Figure 3:**
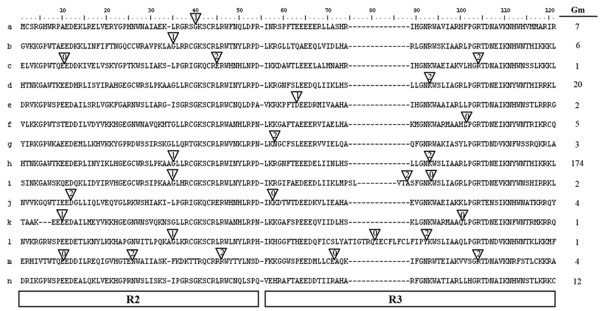
** Schematic diagram of intron distribution patterns within the MYB domains of soybean MYB (GmMYB) proteins.** Alignment of MYB domains is representative of 14 intron patterns, designated a to n. Locations of introns are indicated by white triangles, and the number within each triangle indicates the splicing phases of the MYB domain sequences: 0 refers to phase 0; 1 to phase 1; and 2 to phase 2. The number of GmMYB proteins with each pattern is presented on the right. The correlation of intron distribution patterns and phylogenetic subfamilies is provided in Figure 2 and Additional file 3.

The observed intron distribution, positions, and phases within each subfamily are shown in Figure 3 and Additional file 3. In order to determine the numbers and positions of exons and introns within each soybean MYB gene, we compared the full-length cDNA sequences with the corresponding genomic DNA sequences. We observed that most of the coding sequences of the MYBs were disrupted by introns. By contrast, only 5% of MYBs had no introns in the MYB coding region (Figure 3, pattern n); the remaining genes had up to 14 introns on the basis of their relative positions and phases.

It was previously reported that most *Arabidopsis* MYB family genes possess two introns and that the number of introns in the DNA-binding domain ranges from zero to three [32,33]. In the present study, we found a higher number of intron types in GmMYBs (ranging from one to five in the DNA-binding domain), which could be grouped into 14 patterns of intron presence and positions (Figure 3, patterns a to n). Approximately 74% of soybean genes exhibited pattern h, which has a highly conserved splicing arrangement of three exons and two introns in the R2R3-MYB domain (Figure 3). Besides the typical h pattern, the remaining MYBs had altered splicing sites and showed the remaining 13 patterns (Figure 3). Either of the two introns was absent in 17% of GmMYBs (e.g., subfamily C5, C16, C29, and C30), whereas only 4% of GmMYBs contained more than two introns in the MYB domain.

Remarkably, genes in the same subfamily generally showed the same intron pattern, with the position of the intron almost completely conserved within most subfamilies. This finding constitutes an independent criterion for testing the reliability of our phylogenetic analysis. For example, the subfamily C42 lacked introns 1 and 2; subfamily C5 lacked intron 2; subfamily C30 lacked intron 1; and subfamily C4 retained the typical splicing sites (Figure 2b and Additional file 3). Interestingly, in *Arabidopsis*, most MYBs contain no more than two introns in the MYB domain, except AtMYB88 and AtMYB124 in the ‘guard cell division restriction’ clade, which have a complex exon/intron organization (10 and 11 introns, respectively) [33]. However, in soybean, four homologous genes of AtMYB88 and AtMYB124 (GmMYB051, GmMYB067, GmMYB091, and GmMYB138) were detected and classified in subfamily C46. In common with AtMYB88 and AtMYB124, the GmMYBs in this subfamily also contained a complex exon/intron organization in the entire ORF (10–12 introns). Members of this group contain up to five introns in the MYB domain, which not only supports their close evolutionary relationship, but also indicates the conservation of this intron pattern (pattern m) in evolution. Remarkably, despite the high number of introns in the MYB domain, the phase of the introns is almost conserved (Figure 3 and Additional file 3). This indicates that it is not an occasional mutation event, but a conserved pattern in the evolution of plant MYB transcription factors.

We further observed that the splicing sites of introns were generally located in the R2 and R3 repeats, and that the amino acids close to the splicing sites were highly conserved (Figure 3 and Additional file 2). The most common amino acid in the splicing sites of intron 1 was AG-L in the R2 repeat, whereas the insertion site of intron 2 in the R3 repeat was LGN-WS, typified by pattern h (74% of soybean genes). With the exception of pattern n (no introns), only 13% of the 244 R2R3-MYBs had varied intron positions ( Additional file 3, subfamilies C39 to C47). Multiple sequence alignment analysis showed that one or more of the conserved amino acid residues near the insertion site had been mutated or deleted in the corresponding sites within the MYB proteins (e.g., subfamilies C39, C40, and C43; Additional files 2 and 3). This result suggested that these sites play important roles in the splicing of introns. Interestingly, the major splicing sites of introns were located very close to the conserved positions of each repeat ( Additional file 2). Furthermore, although the number of the introns in the entire ORF regions ranged from one to 14, and the length was variable, all of the introns were typical type I (GT-AG-intron). This may indicate that the intron types of soybean MYB family genes are highly conserved.

In addition to splicing sites, we investigated the intron phase in the MYB domain with respect to codons. The splicing of each intron is thought to occur in one of three phases: phase 0, phase 1, or phase 2, depending on whether the splicing occurs between codons. In phase 0, splicing occurs after the third nucleotide of the first codon; in phase 1, splicing occurs after the first nucleotide of the single codon; and in phase 2, splicing occurs after the second nucleotide [46]. Interestingly, the phases of introns within the same subfamily were almost conserved in the MYB domains (Figures 2 and 3). All of the genes within the same subgroup not only had similar exon patterns, but also exhibited conserved intron phases of the exons; moreover, the gene structures were conserved in soybean and *Arabidopsis*. For instance, subfamilies C4, C7, and C10 (with pattern h) contained three exons, and phase 1 and phase 2 introns in the R2 repeat and R3 repeats, respectively. Similarly, the introns in subfamilies C29, C30, and C42 were phase 1, phase 2, and phase 0, respectively. Our study further revealed a significant excess of phase 1 and 2 introns, and also an excess of nonsymmetrical exons, in the MYB domain. Among the 252 soybean MYB domains analyzed, 79% had phase 1 introns, 93% had phase 2 introns, and only 10% had phase 0 introns. Moreover, the phases in the same sites were almost conserved (Figures 2 and 3). Together, these findings indicate that the splicing phase was highly conserved during the evolution of MYB genes, thus further supporting our subfamily designation (Figure 2b and Additional file 3).

The exon length of the MYBs was highly conserved within each subfamily. Exons 1 and 2 appeared to be more restricted in length, whereas exon 3 was the most diverse in size. In general, exons 1 and 2 were very similar in length (exon 1, 133 bp; exon 2, 130 bp) and were also highly conserved (exon 1, 27% occurrence; exon 2, 68% occurrence). Exon 3 coded the last region of the R3 repeat and the C-terminal regions of the MYB protein. The diverse length of this exon could facilitate the gaining of new or cooperative functional motifs and domains, which may account for the functional divergence between MYB homologs in plants. Interestingly, the exon lengths observed for the R2R3-MYB superfamily in soybean coincided with those documented for *Arabidopsis* and grape [33]. The restricted exon lengths in the MYB domain explain the highly conserved nature of the MYB domain in the plant kingdom during species evolution. Our findings indicate that the introns in the MYB domain may play an important role in the evolution of the MYB gene family by means of unknown mechanisms.

Strikingly, in terms of intron position, length and phase, the soybean gene structures revealed in the present study were consistent with the phylogenetic subfamilies of *Arabidopsis* and rice defined by Jiang et al. [32]. This finding provides a further clue to the evolutionary relationships among plant MYB genes.

### Chromosomal distribution and duplication events among soybean R2R3-MYB genes

**Figure 4:**
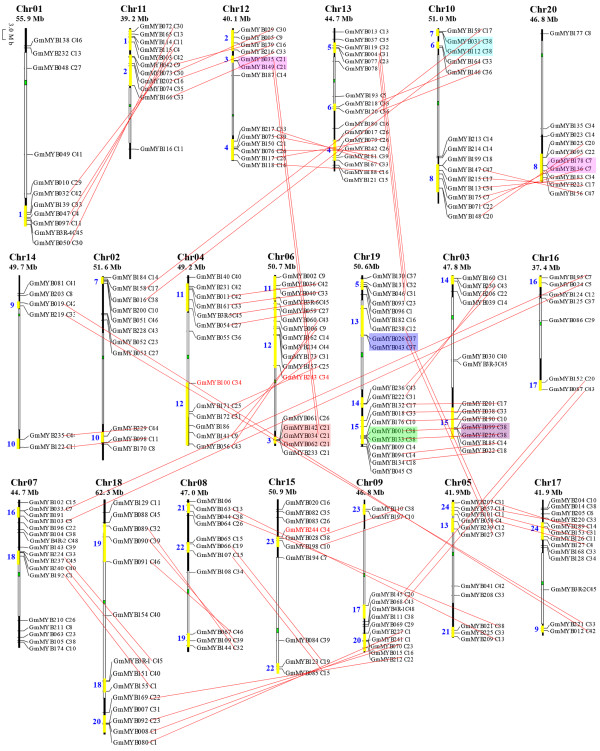
** Chromosomal locations, region duplication, and predicted cluster for soybean MYB genes.** Chromosomal positions of the MYB genes in soybean are mapped on the basis of JGI soybean Genome version 7.0. The chromosome number is indicated above each chromosome. The scale is in megabases (Mb). The number below the chromosome name indicates the length. The phylogenetic category of each gene (Figure 2) is indicated by the subgroup number. Each pair of duplicated MYB genes is connected with a red line. Connecting lines mark the specific cases in which there is a strong correlation between duplicated genomic regions and the presence of MYB genes with closely related predicted amino acid sequences. Colored boxes indicate groups of gene clusters with paralogous and syntenic genes on the chromosomes. Yellow bars on the chromosomes and blue numbers beside the bars indicate the 24 predicted duplication regions. The green and white bars on the chromosomes indicate the centromeres and pericentromeres, respectively.

To determine the genomic distribution of the MYBs, we searched the soybean genome database using the DNA sequence of each soybean MYB gene and BLASTN. Genome chromosomal location analyses revealed that soybean MYBs were distributed on almost all chromosomes. Although each of the 20 soybean chromosomes contained MYBs, the distribution appeared to be uneven (Figure 4). In total, six GmMYBs were present on chromosome 14; seven on chromosome 16; 10 on each of chromosomes 1, 5, 9, 15 and 20; 11 on each of chromosomes 2, 3, 8, 11, and 17; 12 on each of chromosomes 4 and 18; 13 on chromosome 12; 14 on chromosome 10; 15 on each of chromosomes 6 and 7; 16 on chromosome 13; and 19 on chromosome 19. The gene density per chromosome was uneven, ranging from 0.3% to 0.7%, with the highest density observed on chromosome 19 and lowest density on chromosome 14 (Figure 4). On average, one R2R3-MYB gene was presented every 2.5 Mb. Relatively high densities of MYBs were observed in some chromosomal regions, including the top and bottom of chromosomes 3, 4, 5, 6, 7, 8, 10, 12, 13, 18, and 19; the top of chromosomes 1, 9, and 20; and the bottom of chromosomes 2, 11, 14, 15, 16, and 17. By contrast, almost all central chromosomal regions lacked MYBs, including the centromere and the pericentromere regions (Figure 4).

Gene duplication has long been recognized to occur throughout plant evolution, thereby contributing to the establishment of new gene functions and underlying the origins of evolutionary novelty [47,48]. The origin of multigene families is attributed to gene duplication that arose from region-specific duplication or genome-wide polyploidization. This mechanism has been proved to be a prominent feature of plant genome evolution. To detect a potential relationship between GmMYBs and genome duplications, we identified approximately 92 pairs of GmMYBs that were highly similar paralogs in the same subclades and shared a high degree of identity through their protein sequences. For example, the entire protein sequences of GmMYB005 and GmMYB042 shared 92% similarity, whereas those of GmMYB058 and GmMYB127 were 94% similar (Figure 4, linked with red lines). These genes represent approximately 75% (184 of 244) of the soybean MYBs, which supported the hypothesis that they evolved from putative soybean genome duplication events. In addition, multiple pairs linked each of at least 24 potential chromosomal/segmental duplications (Figure 4, pairs of bars with numbers 1–24), such as the large sections of chromosomes 1 and 11. A genome duplication event in soybean occurred approximately 13 million years ago and resulted in a highly duplicated genome [31]. Consistent with this, our results showed that many predicted GmMYBs had paralogous counterparts in syntenic regions of related chromosomes (Figure 4), which indicated that segment duplication is a major driver of gene expansion in soybean.

In the present study, intrachromosomal duplication was also observed in the soybean genome. A series of tandem gene duplications was also indicated to contribute to increasing the number of R2R3-MYB family genes, as evidenced by the fact that some closely related GmMYBs in a single cluster were physically located near to each other on a given chromosome with no intervening annotated gene: for example, GmMYB099/GmMYB226 at the bottom of chromosome 3, GmMYB031/GmMYB112 at the top of chromosome 10, and GmMYB026/GmMYB043 at the top of chromosome 19 (Figure 4, marked with colored boxes). In total, about 5% (13 of 244) of GmMYBs were involved in tandem duplication. In general, the tandem repeat in soybean involved duplicate MYB-encoding genes, with the exception of a triplicate repeat (Figure 4, chromosome 6; GmMYB034/GmMYB062/GmMYB142). Moreover, three specific genes (GmMYB100, GmMYB243, and GmMYB244) contained only the DNA-binding domain (with no C-terminal region), which suggested possible function loss for these genes. The amino acid sequences of GmMYB100 and GmMYB243 were identical; their corresponding cDNA and DNA sequences were also very similar (99.7% and 99.5%, respectively), except for a few discontinuous mutations in the C-terminal region. The amino acid sequence of GmMYB244 was highly similar to those of GmMYB100 and GmMYB243, except for a single-site mutation in the C-terminal region. Their high similarity suggested that these three genes arose from a recent duplication event, thereby providing strong evidence that gene duplication has made an important contribution to soybean MYB gene expansion.

It is likely that different MYBs evolved after duplication of their DNA-binding domain, subsequently by a series of synonymous and/or non-synonymous mutations in the ORF (especially in the C-terminal region), to generate new functions during evolution. Together, our findings indicate that entire genome duplication, segmental duplication, and tandem gene duplication all contributed to R2R3-MYB gene expansion in soybean. The number of tandem duplications was much fewer than the number of genome and/or segment duplications, therefore the latter might have been the main contributor to MYB gene expansion in soybean.

### Conserved motifs outside the MYB domain

Interestingly, the relative spatial location of the plant MYB domain is variable, and may be in the N-terminal, middle, or C-terminal regions of the protein. In typical R2R3-MYB proteins, the MYB domain is usually located close to the N-terminal region, and the very short region before the MYB domain appears to be less conserved between different subfamilies. Consequently, no motifs have been detected in these regions throughout the plant MYB gene family. By contrast, C-terminal regions outside the MYB domain generally contain many specific, functionally important domains or motifs involved in transcriptional activity and protein–protein interactions. Proteins within a subfamily that share the same motifs are likely to share similar functions.

Using MEME, we searched for conserved motifs in the soybean MYB proteins of each group. Most members of a subfamily shared one or more motifs outside the MYB domain, which provided further support for definition of the subfamily. We identified a total of 50 conserved motifs in the C-terminal regions (Table 2), ranging in size from 18 to 250 amino acids; some of these subfamily-specific motifs were previously characterized as defining additional functional properties [4]. Interestingly, except for motif 1, which directly followed the R3 repeat and was specifically conserved in some subfamilies (C8, C9, and C11; Figure 2c and Additional file 3), most of the motifs were selectively distributed among the specific clades in the phylogenetic tree. Our findings demonstrate that the protein architecture is remarkably conserved within a specific subfamily.

**Table 2:** Group and sub-group specific conserved motifs

**Motif**	**E-value**	**Consensus sequences**
1	3.60E-146	L[RK]MGIDPVTH[ST]PRLDLLD[IL]SSIL[NRS][SA]S[LFI][YG][NG]
2	1.80E-44	[AHY][ANS][NY][DS][SK]N[FY][DN]D[GD]M[DE]FW[YF]D[IV][FL][TAI][RKS][ST][GN][ED][SI]IEL[LPS]E
3	7.40E-32	[EP][ETD][INTV][MN][PAL][APV][IL]DE[SD]FWSE[VA][LAT][SIV][DS][DY][EN]
4	8.10E-13	[KD][PK][NP]xL[IL]EIP[FW][DE][LPS]D[HPY]D[FI]W[SK][FL][LI]D
5	2.20E-17	PKL[LF]F[SA]EWL
6	1.00E-90	[HIK][KV][AK][PS][SAE]S[PT]STRHM[AV]QWES[AV]R[LV]EAEARLS[RN]E
7	2.80E-58	[SY]W[NP][NT][NS]KT[DC][SP]D[HY]FL[RQ][IL]WNSEVG[EK][SA]FR[DMN][VI][HK][GK]
8	1.90E-27	D[TS][AS]L[QK][LH]LL[DH][FM]P[ID][NS][ND][DM]
9	7.60E-95	[RK]GQWERRLQTDI[NH]MAK[QKR]AL[SC]EALS[PL][DN]K[PA]
10	8.00E-64	[PT][TN][TP][TY][QP][ST][GMT][CTV]YASS[AT][DE]NIA[RK]LL[KQ][GN][WF]MK[ND][TS]PK
11	4.40E-31	[QD][GM][PS][FL]SL[FL]EKWL[FL]D[DE][QA][GAS][CGH]Q[EMQ]
12	1.20E-145	[KT]N[DE][AT]LLS[ST][DEL]G[QP]SK[DNT]AANLSHMAQWESARLEAEARL[VA]RESK[LI]
13	8.30E-22	[IT]T[SV]N[AV][GT][RV][SV][GQS][DQ]LESPTST[LV]S[FE][SN][EN]N[AV]P[MPS]
14	2.00E-48	[DN][LFY]YE[DE]N[KN]NYWN[SN]I[LF]NL[VM][ND][SY]S[PS]
15	3.70E-12	GIG[LP][EQ][KS]DNIRDPL[EV]PNLTSPSHS[IV]TSAKLLNKMATSLPHKV[HY]GLEA[AV]KAVFSKLME
16	3.60E-19	[FQ]VG[GS]CLNALSPFED[GK]PHI[ST]K[IT]IDSPS[GS]PTLMFN[HQ]VTTPSCFSEDNEDNWQCVSSNYVSF[KR][IN]CAA[NS]GG
17	5.40E-33	[LG][GM][AH]Q[APY][LV][VM]NPE[LFI]LKLA
18	4.10E-12	S[VE][LS][SV]TP[SL]S[ST]PTPLNS
19	2.90E-37	[YFIS][VNS][NG][SCG][SNT]TE[DE]ER[DE][STQ][YS][CYG][SD]
20	4.20E-22	[LQ]A[ELR][ALQ][QTV][EQ][LM]AKLQ[YCL][LF][QH][YQ]
21	1.40E-12	[NY][AGV][EG][DG][ANP][SC][ST][YI]W[PS][ED][LH]L[LF][ED]
22	2.30E-18	[EF][EL][MT]WGDFM[DY]EE
23	8.20E-18	KT[QL]VYLPKPIRVKA[ML][YS][LS][QC][RI][TP][DR]
24	7.30E-15	HG[TN]L[QE][QK]LYEEY[FL]QLLN[MV][DE][EHQ][GK][QP][FD]E[LR][ND]SFA[EQ]SLL
25	6.90E-36	[PYL][NG][HFNQ][DQT][NE][ND][LQV][QTE][TA][KN][PI][PK][VIM]QE[TA][LF]FS[SH][TK][CP]PLF[MI][FV]DT
26	1.70E-35	[FL][EG][SP][VN][NK][IM]G[VAL][EG]GD[FM][SYF][VL]P[PS]LE[SN][RV][ST][ICT][ET][SR][ND]
27	1.90E-55	[EIV][ELS]E[RP][CH][PH][DQ]LNL[ED]L[SRT]I[SG][PL]P[SRW]Q[PQ]Q
28	5.80E-38	ERCPDLNLEL[RT]ISPP[RW]QQQ
29	3.90E-21	CF[AV]C[SR][LF]G[LM]HN[SG][KM][DE]C[SR]
30	3.20E-29	[LF]DMK[GK]I[IM][ANS]LLE[DE][ND]NHRVP[SY]IS
31	2.00E-25	M[HY][APQ][NA][TC][ST][EP][ER][QK]GY[FL]Y[SN]M[FV][NI][PV][NF][CD]
32	5.40E-12	[WF][EG][LV][GMR][SK]SPYE[TN]RI[LS]DWI[AS]E[LI][QS][NT][GD][YQ][GS][ED][AKN][EN]L[ES][QE]D[CH][NS]S[TN][GS][TC][CS][NE][NY][YN]
33	8.80E-28	NYWS[MI][ED]DIWS[MS]
34	1.00E-46	W[TND][DV]E[NS][MFI][WLC][FL]LQQ
35	2.00E-37	[NS]SGLLD[AD]LLEEA[QEK]A[LM][SA]S[GS][KEG][NK]SK[KS][DR][KR]S
36	1.70E-36	GHRA[LW][AG][TD]HKKEAAWRL[SR]R[VL]ELQLESEK[TA][NC]R[RQ]REK[IM]EE[FI]EAK[IM]KAL[QR]EE[EQ][KL][AN]A[LM][GE][RK]IE[AG]EYREQL[ADV][AG]LRRDAE[NA]K[ED]QKLA
37	6.80E-20	[LV][FHY]RPV[AP]R[VLM][SG][AS]F[SN][VAI][YC][NHK]
38	5.20E-19	[CDY][CDGSY][ED][PQR][FIMQS][VI]P[SHQ][KQT]CG[HG]GC[CS]
39	7.10E-26	[EA][LF][IA][SA]I[AS][TN][DE][LI][NGS][NS][ILT]AW[IL][KR]SG[LK][ED]
40	1.70E-55	[ET]T[SA]SS[SE]DDP[PA]TSLSLSLPG[VAF]D
41	1.90E-124	F[SG][GA]EF[ML][AT]V[MV]QEMI[RK]KEVRSYM[SAE]x[ML][QE][QR][GN]NG
42	3.40E-33	[PKV][RG][PN][NS][IL]L[EQ][DN]YI[KR]S[IL][TNE][ILQ][NI]
43	2.50E-150	[RM][LV][SI]RVELQLESEK[AT][GN]RRREK[IM]EE[FI]EAKIKAL[QR]EE[EQ][TKL]AAL[DG]RIEAEYREQL[AD][AG]LRRDAE[NS]KEQKLAEQW[AD]AKHLR[LF]T[KR][FL]LEQ[LSV][GD][CT][RI]
44	4.10E-109	RIGMTPTRDGFSFSMTPKGTPLRD[AE]LHINEDMNMHDSTKLELQRQADMRRSLRSGLGSLPQPKNEYQIVM[PQ]PV[LP]EDAEEPEEKIEEDMSDRIAREKAEEEARQQALLRKRSKVLQRELPRPPTASLELIRNSLMRTD[GV]DKSSFVPPTSIEQADEMIRRELL[ST]LLEHDN[AG]KYPLD[DE]KVIKEKKKGAKRAVNGSAVPVIEDF[EQ]EDEMKEADKLIKEEALYLCAAMGHEDEPLDEFIEAHRTCLNDLMYFPTR
45	4.10E-171	A[QE]NNKIQGTFLKKDDPK[IV][NST]ALMQQAELLSSLA[LQ]KV[DN]A[ED]N[MT]DQS[LM]ENAWKVLQ[ED]FLN[RK][TS]KE[SN]D[IL][PF][RG][YQ][KG]IPD
46	1.00E-48	[LF]QLV[DE][LF]KDL[IL][EA]DL[KR]S[GS][NS]E[ED][GI]Q[AP][CS]WRQ[MPV][DE]L[YH
47	1.80E-123	[EK]FSSP[LI]QVTPLFRSLA[AD]GIPSPQFSESER[SN]FL[MRL]KTLG[MI]ES[PS]S[ILP][NC]PS[AV]N[PS]S[QK]PP[PL]CKR[AV]LL[IPH]
48	4.90E-79	GIDYNAEIPFEKR[PA]P[PA]GF[FY]D[VT][TA]DEDRP[VA][ED]Q[PV][QK]FPTTIEELEGKRR[VA]DVEA[QH]LRKQD[IV]A[KR]NKIAQRQDAP[SA]AIL[HQ]ANKLNDPE[TV]VRKRSKLMLPPPQISD[QH]
49	3.50E-92	KGDAIMMEAENLARLR[ED]SQTPLLGGENPELHPSDF[NST]GVTP[KR]KKEIQTPNPMLTPS[AM]TPG[GA]AGLTPRIG[ML]TP[TS]RDG[FS]SFSMTPKGTP[LF]RD[EA]LHINEDM[ND]MH
50	6.20E-59	I[ED]DF[DEQ]E[DN]E[ML][KQ]EADK[LM]IKEE[AG][LK][YF]LC[AV][AS]MGHE[DN][EK][PT]LD[ED]F[IV]EAH[RN]TC[LV]NDLMYFPTR[NS]AY[GE]LSSVAGN[MA][ED]K[LV][AT]A[LF]Q[NE]E[FM]ENVR

The schemes of protein motifs of individual members of the MYB family revealed the structural similarities among proteins within subfamilies (Figure 2a and Additional file 3). Although the functions of most of these conserved motifs have not been characterized, it is likely that some play important roles in the transcriptional regulation of target genes. For instance, motifs 7 and 8, which are conserved among members of subfamily C9 (Figure 2c), have been characterized in AtMYBs as a C2 repressor motif and zinc finger. Members of this group possess the C1 and C2 motifs, which are known to participate in bHLH interactions and promoter repression, respectively. In the present study, we identified three orthologous GmMYBs that contained the same conserved motifs in the C-terminal region, which have been shown to function as a repression domain. It is likely that these orthologous MYBs also function as repression factors in soybean.

In *Arabidopsis*, three atypical MYB genes (AtMYBCDC5, AtMYB3R-like, and AtMYB4R1), which encode MYB proteins with two or more repeats, are distantly related to the typical R2R3-MYB proteins [4,27,42]. AtMYBCDC5 contains an MYB domain, consisting of two repeats, that shows very low homology (approximately 31% identity) to the typical R2R3-type MYB domain, and a very long C-terminal region (more than three-fold longer than the typical R2R3-type MYB gene). In soybean, we observed two genes (GmMYB147 and GmMYB156) with high homology to AtMYBCDC5 ( Additional file 3; subfamily C47). Remarkably, although the length of the coding region and the intron pattern outside the MYB domain were different, the intron phase and splicing site within the MYB domain were almost identical (Figure 2b). A MEME search using the amino acid sequences of these three atypical MYB proteins identified three unexpectedly large conserved motifs ( Additional file 3; motifs 48, 49, and 50) in the C-terminal region of this subfamily. This finding may facilitate the identification of functional characteristics in these types of MYBs. However, their biological functions remain to be elucidated. We also observed two orthologous genes to AtMYB4R1, which is a putative MYB protein containing four R1R2-like repeats ( Additional file 3; subfamily C48). Although the region before the MYB domain is likely to be longer than that of typical MYB genes, it appears not to be conserved in this subfamily. No subfamily-specific conserved motifs were detected at the N- or C-termini. Moreover, no homologous AtMYB3R-like gene was identified in the soybean genome; this may reflect gene loss during evolution of the soybean genome.

### Expression pattern of R2R3-MYB genes in soybean and *Arabidopsis*

Transcription factor genes comprise a substantial fraction of all eukaryotic genomes. On the basis of the type of DNA-binding domain that they encode, the majority of transcription factor genes can be grouped into a handful of different, often large, families. Functional redundancy is not unusual within these families; thus, the proper characterization of a particular transcription factor gene often requires its study in the context of a whole family. By forming intricate networks through protein–protein interactions, and also at the transcriptional level, transcription factors control the expression of the genome. Ultimately, therefore, their functions cannot be understood without considering their activities at a genome-wide scale. It has been noted previously that many transcription factor gene families exhibit great disparities in abundance among different eukaryotic organisms, and that some families are lineage specific.

The recently developed RNA-Seq web-based tools, which include gene expression data across multiple tissues and organs, allow characterization and comparison of the gene transcriptome in soybean [30]. Consequently, distinct transcript abundance patterns are readily identifiable in the RNA-Seq Atlas data set for all 244 GmMYBs. The gene expression pattern can provide important clues for gene function. We therefore obtained expression information for each clade, and compared the expression profiles of MYB transcription factor subfamilies of soybean with their *Arabidopsis* counterparts in root, leaf, flower, and seed tissues using SoySeq and AtGenExpress data. We subsequently summarized these expression profiles against the phylogenetic tree (Figure 2d and Additional file 3). In order to assess the potential role of MYBs in legume-specific processes, we also investigated the expression of the soybean R2R3-MYB gene family during nodulation.

In common with genes that encode transcription factors, many of the GmMYBs exhibited low transcript abundance levels, as determined by the RNA-Seq Atlas analysis. After integration of the two data sets, we observed that most of the genes had very broad expression spectra. However, five AtMYBs and 32 (13%) GmMYBs lacked expression information, which possibly indicated that these were pseudogenes or were expressed only at specific developmental stages or under special conditions. Next, we examined data from a second soybean transcriptome atlas [49], which is an integrated transcriptome atlas of a variety of soybean tissues. The results showed that all of the 32 GmMYBs were predicted with high confidence in soybean tissues (data not shown), which demonstrated that these genes were expressed under highly restricted conditions.

We observed that accumulation of R2R3-MYB gene transcripts was associated with different tissues, and the expression pattern of each R2R3-MYB gene member differed (Figure 2d and Additional file 3). In soybean, only a small number (23 of 244; 9%) of the analyzed MYBs were constitutively expressed in all of the four tissues tested, which suggested that GmMYBs play regulatory roles at multiple developmental stages. By contrast, most GmMYBs showed preferential expression. RNA-Seq Atlas data revealed that the majority (157 of 244; 64%) of GmMYBs exhibited transcript abundance profiles with marked peaks in only a single tissue. This result suggests that these R2R3-MYB proteins function as plant-specific regulators and are limited to discrete cells, organs, or conditions. Approximately 99 of these 244 (41%) GmMYBs showed the highest transcript accumulation level in flower tissue, 82 (34%) showed the highest transcript accumulation in root tissue, 41 (17%) showed the highest transcript accumulation in leaf tissue, and 60 (25%) showed the highest transcript accumulation in seed tissue (some genes showed equal transcript accumulation levels in more than two tissues). The wide expression of these genes suggests that the MYBs from soybean and *Arabidopsis* are involved in the development of all organs or tissues under specific conditions.

Although the expression pattern of MYBs varied between soybean and *Arabidopsis*, we also observed conserved expression profiles. In addition to groups of genes that exhibited similar transcript abundance profiles but were relatively phylogenetically distinct, several phylogenetic clades shared, to a large extent, the same transcript abundance profile. For example, in subfamily C1, most of the AtMYBs and GmMYBs were expressed in flower tissue, which indicated their conserved functional role in flower development. The expression of members of subfamily C20, in soybean and also in *Arabidopsis*, was detectable in root tissue, which suggested their conserved roles in root formation. Members of subfamily C23 showed similar expression patterns in flower and seed development, which supported the hypothesis that these genes function in soybean and *Arabidopsis* differentiation (Figure 2d and Additional file 3). By contrast, GmMYBs with high sequence similarity and shared expression profiles represent good candidates for evaluation of gene functions in soybean. Prominent among these clades are those that include MYBs related to the regulation of phenylpropanoid metabolism (subfamily C20).

Transcription factors are not only involved in the activation of plant defense systems, but also play key roles in the control of mutualistic interactions between plant roots and soil microorganisms. For example, nodulation involves the interaction between root and soil bacteria, leading to symbiotic nitrogen fixation, which is mainly restricted to legumes. The first transcription factor shown to be involved in nodulation was the *Lotus japonicus* NIN gene [50,51]. Subsequently, the *Medicago truncatula* ortholog of LjNIN was characterized by screening fast-neutron and Tnt1 transposon-tagged mutagenized populations [52,53]. Other transcription factors critical to the nodulation process have been identified, including GRAS family proteins in *M.truncatula* and *L. japonicus*[54,55], the bZIP transcription factor ASTRAY in *L.japonicus*[56], the AP2-EREBP transcription factor MtERN in *M.truncatula*[57-59] and *L.japonicus*[60], and the ARID transcription factor LjSIP1 in *L. japonicus*[61,62]. In the present study, we observed that 64 of 244 GmMYBs exhibited transcript accumulation during legume-specific nodulation, with 39 (16%) of them showing the highest expression levels in this tissue (Figure 2d and Additional file 1). However, only three of the nodulation-expressed genes (*GmMYB201GmMYB034* and *GmMYB035*) are likely to be nodulation-specific, because no transcript accumulation was detected in other tissues. Moreover, *GmMYB201* has been proved to be nodulation-specific, recently [63]. These results indicated that the MYB transcription factors might have a major role in regulation of legume-specific nodulation. It is interesting that what makes legumes special, such as the trait of symbiotic nitrogen fixation, and how these traits evolved in legumes. However, there are not significant differences in transcription factor gene distribution across transcription factor families in plants which suggest that species-specific traits may be likely dependent on transcription factor gene expression patterns and their functions. Interestingly, in our synteny analysis of *Arabidopsis* and soybean genomes, none of these three nodulation-specific-expressed GmMYBs has ortholog in *Arabidopsis*, which suggests that they may be new functionalization after the divergence of soybean and *Arabidopsis.* These results indicated that MYB genes may be important in the evolutional development of nodule in legumes. Although many of the GmMYBs showed high transcript accumulation during nodulation, the lack of corresponding orthologs involved in this process means that the functional characteristics of the GmMYBs in legume-specific nodulation remain to be elucidated.

Given that the soybean MYB gene family is so large, a key question is whether the various paralogs show similar expression patterns suggestive of functional redundancy. As shown in Figure 2 and Additional file 3, most pairs of paralogs in soybean shared similar expression patterns and also similar expression levels, and thus showed functional redundancy. By contrast, the remaining pairs exhibited significant divergence of expression, which supported the notion that the expression of paralogs can diverge significantly after duplication. However, the orthologs generally show similar expression patterns. Overall, the expression of these genes in widespread tissues suggests that the MYBs from soybean and *Arabidopsis* are involved in the development of all organs or tissues under specific conditions, such as legume-specific nodulation. In addition, the expression of MYBs in *Arabidopsis* or soybean supports conservation of functions for gene orthologs across plant species. Discrepancies in the expression patterns of homologous genes may indicate a divergence in function. Nevertheless, the functions of MYBs appear highly, but not absolutely, conserved across plant species. Although the functions of most GmMYBs are unknown, our phylogenetic and expression analyses provide a solid foundation for future functional studies in soybean and *Arabidopsis*.

### Putative functions of soybean R2R3-MYB transcription factors

Phylogenetic analysis allowed identification of putative orthologous and paralogous MYBs. It was suggested previously that orthologous genes usually share similar functions and are clustered in the same clades and subclades, whereas paralogous genes generally display different functions, which indicates that closely related MYBs can recognize similar target genes and may possess functional redundancy. Thus, analysis of orthologous and paralogous genes is essential to comparative genomics.

Currently, the most extensive annotative evaluation of plant MYBs was obtained from studies of *Arabidopsis*. In order to update functional clades that could also be present in soybean subfamilies, we constructed a phylogenetic tree to define MYB families of orthologs from different species, based on the well-characterized R2R3-MYB genes in *Arabidopsis* and some other plant species (Figure 2a and Additional file 3). Within each subfamily, we identified particular clusters of paralogous and orthologous genes, which were helpful for characterization of each subfamily. For example, subfamily C1 consisted of four AtMYBs (AtMYB75, AtMYB90, AtMYB113, and AtMYB114,), 10 MYBs from other plant species (PhAn2; NtAn2; LeAn1; MdMYB10; VvMYBA1 and VvMYBA2; PyMYB10; ROSEA1; GhMYB10; and IbMYB1), and seven GmMYBs (Figure 2a). These included gene members that contain motif 2 in the C-terminal region and are likely to have a consensus intron pattern. Although the functions of the GmMYBs in this subfamily are unknown, the remaining genes are known to be involved in anthocyanin biosynthesis. Thus, it is reasonable to hypothesize that the GmMYBs may also play a role in the anthocyanin biosynthesis pathway. Subfamily C10 contains seven GmMYBs, three AtMYBs (AtMYB11, AtMYB12 and At MYB111) and five MYBs from other plant species (y1; P1; P2; VvMYBF1; and SlMYB12. Except for the GmMYBs, the other genes are well known to play crucial roles in the regulation of flavonol biosynthesis (Figure 2a) [16]. Thus, the GmMYBs in this subgroup might play important roles in the flavonol biosynthesis pathway. Similar results were observed for the MYBs in subfamily C38, among which the MYBs from other plant species (AtMYB2, AtMYB62, AtMYB78, AtMYB108, AtMYB112 and AtMYB116; TaPIMP1) are known to be involved in defense-related pathways, such as drought, salt, and pathogen response [44]. The genes of subfamily C39 are involved in GA-induced responses (AtMYB33, AtMYB65, AtMYB81 and AtMYB101; LtGAMYB; HvGAMYB; and OsGAMYB), whereas subfamily C40 genes (AtMYB91; AmPHAN; LePHAN; ZmPHAN; and WRS2), including four soybean orthologs, are suggested to be involved in rough-sheath development (Figure 2a and Additional file 3). Similarly, eight GmMYBs with homology to four AtMYBs (AtMYB44, AtMYB70, AtMYB73 and AtMYB77) in subfamily C42 were indicated to respond to abiotic stress [42].

In general, most plant MYBs are positive regulators of transcription. For example, AtMYB12 activates the phenylpropanoid biosynthetic pathway by up-regulating the expression of chalcone synthase (*CHS*), flavanone 3-hydroxylase (*F3H*), flavonol synthase (*FLS*) and, to a lesser extent, chalcone flavanone isomerase (*CHI*[15]). C1 in maize [12], and its orthologs PcMYB1 in *Petroselinum crispum*[64] and AtMYB111 in *Arabidopsis*[16], positively regulate flavonoid biosynthesis by controlling the expression of *CHS*. Transcriptional regulation of gene expression is mediated not only by activators, but also by the action of repressors. Previous studies indicate that some MYB genes act as transcriptional repressors or weak activators. For example, in *Arabidopsis*, AtMYB4 regulates the accumulation of UV-protective sinapate esters by repressing the expression of the gene encoding the key enzyme cinnamate-4-hydroxylase (*C4H*[19]). Similarly, AtMYB32 represses expression of the *COMT* gene in *Arabidopsis*[65], whereas the strawberry FaMYB1 transcription factor suppresses anthocyanin and flavonol accumulation in tobacco [66]. Further studies have demonstrated that the C-terminal region of these MYBs often possesses repression motifs. For example, the C2 motif repressor clade possesses the conserved C2 motif (pdLNLD/ELxiG/S) at the C-terminus, and is known to be involved in the repression of transcription. The present study revealed that three GmMYBs (GmMYB002, GmMYB005, and GmMYB042) had the same repression motif in their C-terminal regions. These genes were classified in subfamily C9, which indicated that they are likely to act as repressors.

Combinatorial interactions between transcription factors are pivotal to the regulation of eukaryotic gene expression. The combinatorial regulation of the flavonoid biosynthetic pathway by the bHLH-MYB-WD40 complex is well characterized. These interactions can either modulate transcription factor activity or contribute to the biological specificity of factors with very similar DNA-interaction motifs. In maize, the MYB protein C1/Pl was reported to depend on the interaction with R/B (bHLH protein) for its regulatory function in anthocyanin biosynthesis [12]. In *Arabidopsis*, TT2, TT8, and TTG1 (which encode MYB, bHLH, and WDR proteins, respectively) regulate expression of several flavonoid structural genes [67,68]. Similar regulatory factors that control phenylpropanoid biosynthesis have been isolated from other species [69,70]. Further investigations revealed that four residues in the first helix (all four residues must be present) and two additional residues in the second helix of the C1 R3 repeat are necessary for the interaction with R in plant cells [71]. Interestingly, in the present study, we observed that up to 10 GmMYBs had all six residues in the same location. Although these clustered in different clades within the MYB subfamily (C4 and C6), and appeared to have different target genes, we propose that these genes have a combinatorial regulatory mechanism to regulate expression of soybean target genes.

The 3R-MYB genes are present predominantly in vertebrates; however, members of this three-repeat MYB gene family have been detected also in some plant lineages, including *ArabidopsisVitis vinifera*, and *Populus trichocarpa*[26,72,73]. The fact that only five paralogous genes exist in each of these species indicates that the number of plant 3R-MYB genes is strictly limited. Interestingly, we detected six 3R-MYB *Arabidopsis* orthologous genes in the soybean genome, which implied that soybean 3R-MYBs may be undergoing expansion ( Additional file 3, subfamily C45). Our findings suggest that the 3R-MYB genes represent an ancient and evolutionarily conserved plant gene family. Moreover, in contrast to the functional diversity of the R2R3-MYB proteins, it appears that 3R-MYBs fulfill a number of core cellular functions, which are evolutionarily conserved [72-74]. Thus, it is credible to hypothesize that soybean 3R-MYBs may have the same function.

## Conclusions

MYB genes are widely distributed among higher plants and comprise one of the largest groups of transcription factors in the plant kingdom. However, the specific roles of most plant MYB genes remain unknown. In the present study, we attempted to elucidate characteristics of soybean MYB gene structures, phylogenetic relationships, chromosomal locations, conserved motifs and expression patterns, as well as their functional diversification, by identifying the probable full complement of GmMYB genes in soybean. We performed extensive analyses of the candidate soybean MYB genes and compared them with those of *Arabidopsis*.

We classified the soybean and *Arabidopsis* MYBs into 48 subfamilies on the basis of their phylogenetic relationships, which were well supported by additional conserved protein motifs, and intron/exon structures. The results of our phylogenetic analysis were in good agreement with those of previous studies [29,32,33]. The majority of subfamilies contained members from soybean and *Arabidopsis*, which indicated that members within a given subfamily have recent common evolutionary origins and that the functions of most MYB genes were conserved during angiosperm evolution. The lack of *Arabidopsis* or soybean family members in some clades suggested that diversification among the two species may have contributed to lineage-specific phenotypic innovations. Analysis of intron/exon structures revealed that the splicing sites, lengths, and phases of most introns were highly conserved in the MYB domains, especially in those within the same subfamily. Thus, the MYB domains may originally have been compact and conserved in size during evolution. Chromosomal distribution, phylogenetic analysis and synteny analysis revealed the presence of genome duplication events in the soybean lineage throughout the soybean genome. Gene expansions were observed as gene pairs or clusters. Therefore, genome duplication events may have led to gene diversification and redundancy during expansion of the MYB gene family. Computational searches were used to identify conserved C-terminal motifs present in the different subfamilies. We observed that most motifs appeared to be specific characteristics of the different subfamilies.

In addition, comparative expression profile analysis of MYBs in soybean and *Arabidopsis* revealed that MYBs may play conserved and various roles in different plant biological processes, indicating a divergence in function, and some MYBs may be involved in soybean seed development and legume-specific nodulation. Taken together, the extensive expression data, together with available functional data for AtMYBs, supported the hypothesis that GmMYBs perform a variety of functions in different tissues, at multiple developmental stages. Although the functions of most plant MYBs remain unknown and many experiments are needed to determine their precise functions, our phylogenetic and expression analyses of the MYB gene family in soybean and *Arabidopsis* establishes a solid foundation for future comprehensive functional analysis of GmMYBs.

## Methods

### Database search for MYB proteins in soybean and *Arabidopsis*

Gene identifiers for 126 *Arabidopsis thaliana* R2R3- and R1R2R3-MYB genes were obtained from Stracke et al. (2001). The corresponding protein sequences were downloaded from The Arabidopsis Information Resource (TAIR; http://www.Arabidopsis.org/). A preliminary search for soybean MYB proteins was performed using BLASTN and BLASTP provided by the Joint Genome Institute (JGI) *Glycine max* version 7.0 website (http://www.phytozome.net/cgi-bin/gbrowse/soybean/). To confirm the obtained amino acid sequences, the putative MYB sequences were examined for the MYB domain using the hidden Markov model of the SMART tool (http://smart.embl-heidelberg.de/[75]) and ExPASy Proteomics Server (http://expasy.org/prosite/[76]). All soybean MYB proteins were manually inspected to ensure that the putative gene models contained two or three MYB DNA-binding domains, and that the gene models mapped to unique loci in their respective genomes. We discarded from the data set redundant sequences with different identification numbers and the same chromosome locus.

On the basis of the results of BLASTP searches in the soybean genome database of JGI (using the predicted proteins of soybean MYB genes), we obtained information on cDNA sequences, genomic sequences, intron distribution patterns, phases, and intron/exon boundaries. The centromeric and pericentromeric regions of soybean chromosomes and the synteny within soybean genome were detected by JGI soybean browser (http://www.phytozome.net/cgi-bin/gbrowse/soybean/). From the results of BLASTP searches in the JGI *Glycine max* Genome Browser version 2.16, we obtained information on the chromosome locations.

### Multiple sequence alignments

Using the Multalin tool (http://prodes toulouse.inra.fr/multalin/[77]) with default parameters, we performed multiple sequence alignments on the obtained sequences of the MYB domains. We manually adjusted the alignments by location of the corresponding amino acids in the MYB motif, and highlighted similar amino acids using BioEdit version 7.0.0 software (Pittsburgh Supercomputing Center; http://www.psc.edu/biomed/genedoc/[78]). We used ClustalX (http://www-igbmc.u-strasbg.fr/BioInfo/[79]) as a secondary method to align sequences and recheck the results. To align the motif common to MYB members, we manually adjusted the alignment using BioEdit.

Nucleotide substitutions levels were calculated using the alignment of MYB domain of GmMYB proteins by Hyphy version 2.0 [80] along with a corresponding NJ phylogenetic tree ( Additional file 6). The HyPhy batch file NucModelCompare.bf with a model rejection level of 0.0002 was used to establish the best-fitting of 203 general time reversible models of nucleotide substitution [80]. The HyPhy batch Quick Selection Detion.bf was used to estimate site-by-site variation in rates.

To compare the evolutionary relationships of soybean and *Arabidopsis* MYBs, the domains of GmMYBs and AtMYBs were aligned with ClustalX.

### Protein motif identification

In order to investigate protein motifs in more detail, we used the MEME version 3.5.7 tool to identify conserved motifs shared among MYB proteins [81]. The following parameter settings were used: distribution of motifs, zero or one per sequence; maximum number of motifs to find, 50; minimum width of motif, six; maximum width of motif, 250 (to identify long R2R3 domains); and motif must be present in all members within the same subfamily. Other options used the default values. Only motifs with an e-value of <1e-20 were retained for further analysis. Subsequently, the MAST program was used to search detected motifs in protein databases [82].

### Phylogenetic analysis

To explore the evolutionary relationships of soybean and *Arabidopsis* MYBs, we constructed a NJ tree from the aligned soybean and *Arabidopsis* MYB domains using MEGA 4.0 (http://www.megasoftware.net/index.html; [83]) with the following parameters: Poisson correction, pairwise deletion, and bootstrap analysis with 1000 replicates. On the basis of the results of previous studies [1,32], we considered the AtCDC5 gene (which contained an atypical R2R3 domain) as an R2R3-MYB protein, and included it in our phylogenetic analysis to determine whether its orthologs exist in the soybean genome. We also included the 4R-MYB protein (which contains two adjacent duplicated R1R2 domains) and the 3R-MYB protein (which contains a typical R1R2R3 domain in soybean and *Arabidopsis*). To validate the results obtained with the NJ method, we performed a MP analysis, and a bootstrap analysis with 1000 replicates, with MEGA 4.0.

In this study, to identify the orthologs, we defined synteny blocks to include conservation of gene content, order and direction between different chromosomes and/or genomes. The cross-genome syntenic relationships between *Arabidopsis* and soybean was identified and cataloged by the plant genome duplication (PGDD, http://chibba.agtec.uga.edu/duplication/[84]), with the available whole genome sequences of these two species.

### Expression analysis of AtMYBs and GmMYBs

We used the Soyseq (http://soybase.org/soyseq/[30]) and AtGenExpress (http://www.weigelworld.org/resources/microarray/AtGenExpress[85]) databases to detect the expression patterns of GmMYBs and AtMYBs. The highest values among the published expression values of the four organs were selected.

## Authors’ contributions

HD carried out the genome search, sequence analysis, phylogenetic analysis, interpreted the results and drafted the manuscript. BF, together with HD, contributed to the genome search and compiled the sequence alignments. SY and LL contributed to the phylogenetic, expression and structure analyses. ZL carried out the synteny, and nucleotide substitution analyses. YH and YT jointly contributed to the conception and coordination of the study, were involved in revising the manuscript, and gave final approval of the version to be published. All authors read and approved the final manuscript.

## Supplementary Material

Additional file 1 Summary of the MYB transcription factor genes in soybean. Click here for file

Additional file 2 Alignment of all MYB proteins used in this study. Click here for file

Additional file 3 **The complete version of Figure 2: Phylogenetic relationships and subgroup designations in MYB proteins from soybean (Gm), *Arabidopsis*(At) and other plants.** Click here for file

Additional file 4 **MP phylogenetic tree of 387 MYB proteins in soybean,*Aribidopsis*and other plants.** Click here for file

Additional file 5 **Synteny blocks of*Arabidopsis*and soybean genomes containing MYB genes** Click here for file

Additional file 6 NJ phylogenetic tree of 244 soybean R2R3-MYB proteins. Click here for file
