# Supplementary material for: Genome-wide analysis of the MYB transcription factor superfamily in soybean
Source: BMC Plant Biol. 2012 Jul 9;12:106. doi: 10.1186/1471-2229-12-106 (PMC3462118; doi:10.1186/1471-2229-12-106)
Supplement: Additional file 2 — Alignment of all MYB proteins used in this study. [file 1471-2229-12-106-S2.pdf]

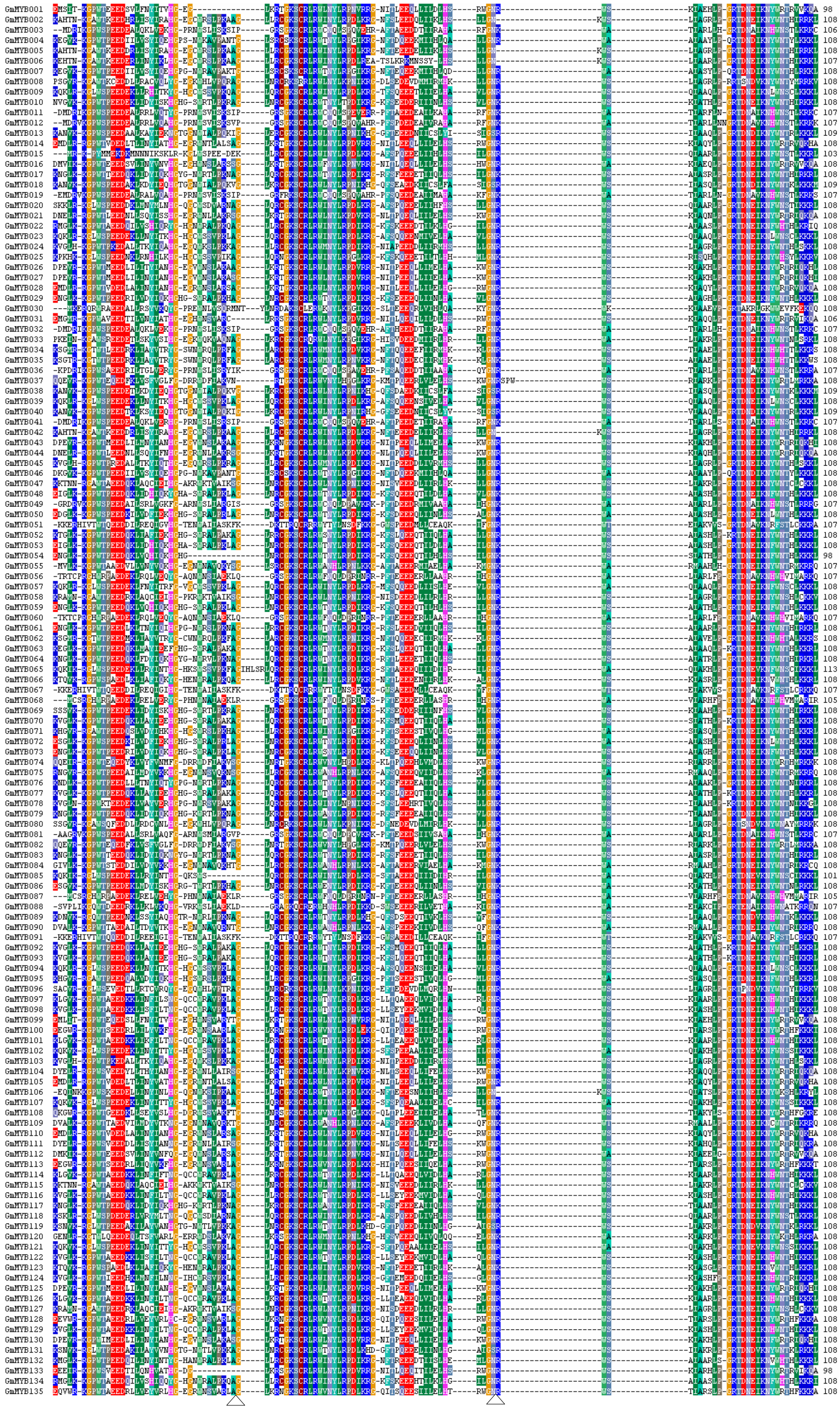
GmMYB001
GmMYB002
GmMYB003
GmMYB004
GmMYB005
GmMYB006
GmMYB007
GmMYB008
GmMYB009
GmMYB010
GmMYB011
GmMYB012
GmMYB013
GmMYB014
GmMYB015
GmMYB016
GmMYB017
GmMYB018
GmMYB019
GmMYB020
GmMYB021
GmMYB022
GmMYB023
GmMYB024
GmMYB025
GmMYB026
GmMYB027
GmMYB028
GmMYB029
GmMYB030
GmMYB031
GmMYB032
GmMYB033
GmMYB034
GmMYB035
GmMYB036
GmMYB037
GmMYB038
GmMYB039
GmMYB040
GmMYB041
GmMYB042
GmMYB043
GmMYB044
GmMYB045
GmMYB046
GmMYB047
GmMYB048
GmMYB049
GmMYB050
GmMYB051
GmMYB052
GmMYB053
GmMYB054
GmMYB055
GmMYB056
GmMYB057
GmMYB058
GmMYB059
GmMYB060
GmMYB061
GmMYB062
GmMYB063
GmMYB064
GmMYB065
GmMYB066
GmMYB067
GmMYB068
GmMYB069
GmMYB070
GmMYB071
GmMYB072
GmMYB073
GmMYB074
GmMYB075
GmMYB076
GmMYB077
GmMYB078
GmMYB079
GmMYB080
GmMYB081
GmMYB082
GmMYB083
GmMYB084
GmMYB085
GmMYB086
GmMYB087
GmMYB088
GmMYB089
GmMYB090
GmMYB091
GmMYB092
GmMYB093
GmMYB094
GmMYB095
GmMYB096
GmMYB097
GmMYB098
GmMYB099
GmMYB100
GmMYB101
GmMYB102
GmMYB103
GmMYB104
GmMYB105
GmMYB106
GmMYB107
GmMYB108
GmMYB109
GmMYB110
GmMYB111
GmMYB112
GmMYB113
GmMYB114
GmMYB115
GmMYB116
GmMYB117
GmMYB118
GmMYB119
GmMYB120
GmMYB121
GmMYB122
GmMYB123
GmMYB124
GmMYB125
GmMYB126
GmMYB127
GmMYB128
GmMYB129
GmMYB130
GmMYB131
GmMYB132
GmMYB133
GmMYB134
GmMYB135

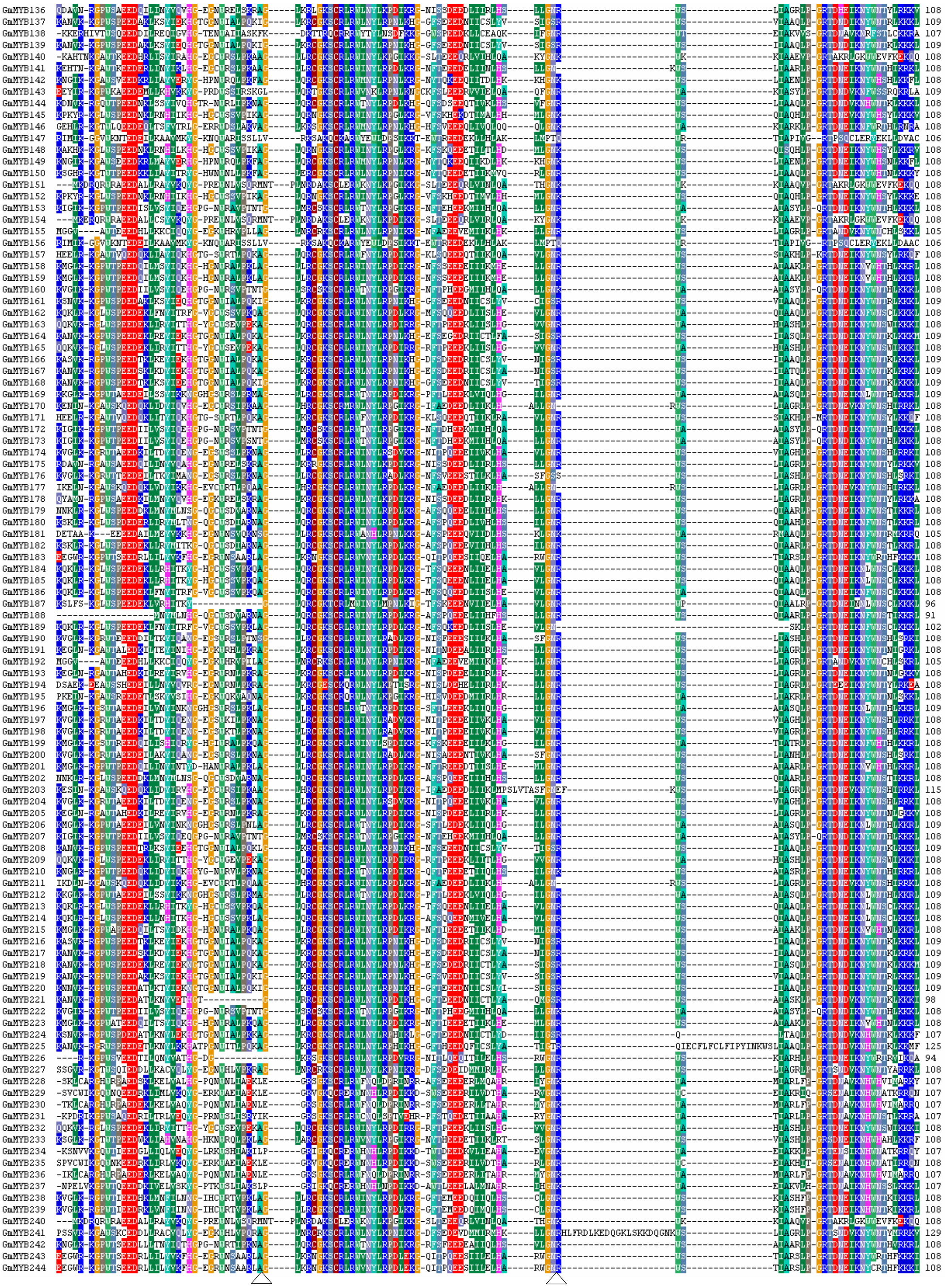

Additional file 2. Alignment of all MYB proteins used in this study.

CLUSTALW amino acid sequence alignment of the 244 soybean MYB domains. The shading of the alignment represents different degrees of conservation among sequences, respectively. The triangles indicate the most conserved intron insertion sites in the MYB domain.
