# Supplementary material for: Genome-wide analysis of the MYB transcription factor superfamily in soybean
Source: BMC Plant Biol. 2012 Jul 9;12:106. doi: 10.1186/1471-2229-12-106 (PMC3462118; doi:10.1186/1471-2229-12-106)
Supplement: Additional file 3 — The complete version of Figure 2: Phylogenetic relationships and subgroup designations in MYB proteins from soybean (Gm), Arabidopsis(At) and other plants. [file 1471-2229-12-106-S3.pdf]

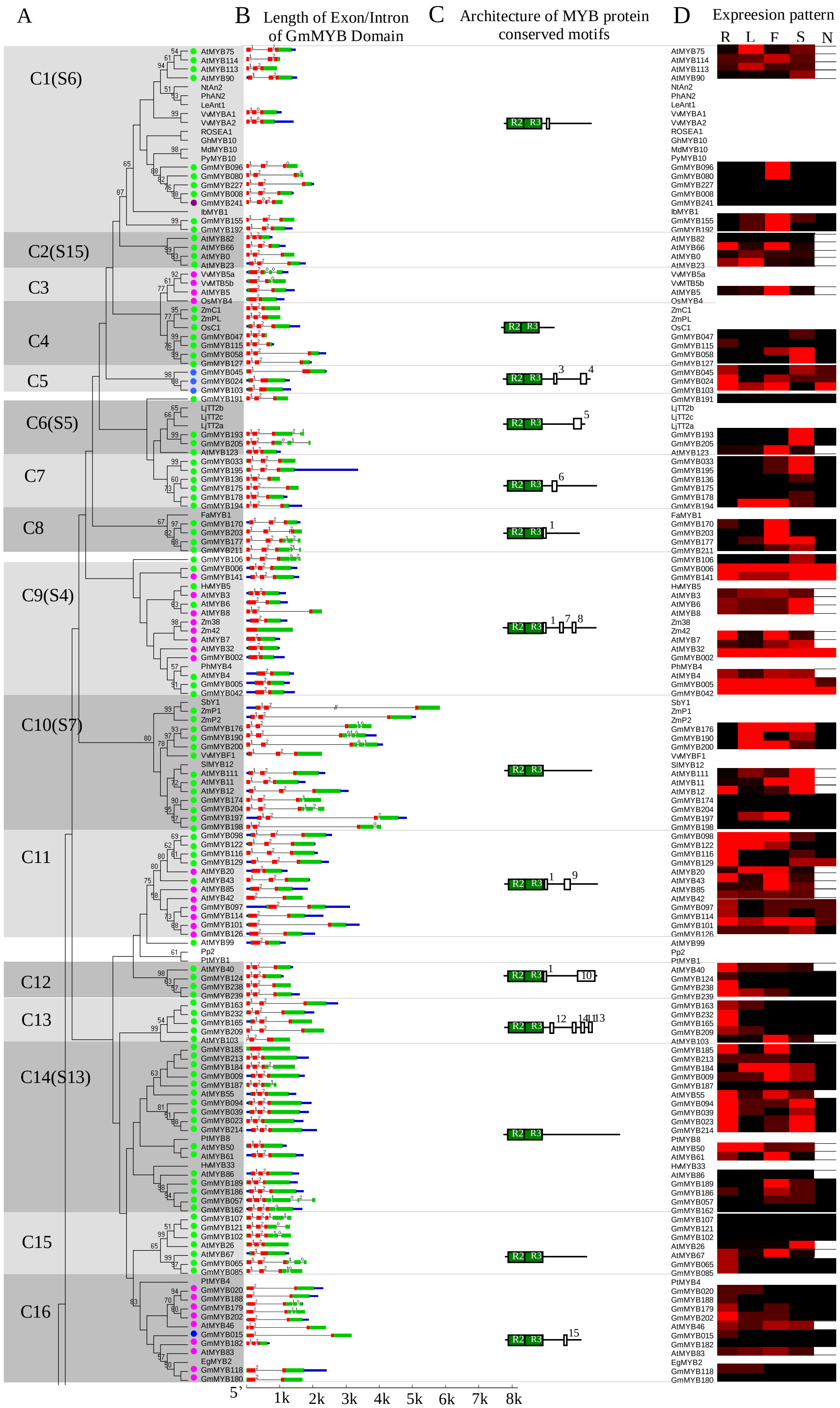

A
B Length of Exon/Intron of GmMYB Domain
C Architecture of MYB protein conserved motifs
D Expreesion pattern
R L F S N
C1(S6)
C2(S15)
C3
C4
C5
C6(S5)
C7
C8
C9(S4)
C10(S7)
C11
C12
C13
C14(S13)
C15
C16
AtMYB75
AtMYB114
AtMYB113
AtMYB90
NtAn2
PhAN2
LeAnt1
VvMYBA1
VvMYBA2
ROSEA1
GhMYB10
MdMYB10
PyMYB10
GmMYB096
GmMYB080
GmMYB227
GmMYB008
GmMYB241
IbMYB1
GmMYB155
GmMYB192
AtMYB82
AtMYB66
AtMYB0
AtMYB23
VvMYB5a
VvMYB5b
AtMYB5
OsMYB4
ZmC1
ZmPL
OsC1
GmMYB047
GmMYB115
GmMYB058
GmMYB127
GmMYB045
GmMYB024
GmMYB103
GmMYB191
LjTT2b
LjTT2c
LjTT2a
GmMYB193
GmMYB205
AtMYB123
GmMYB033
GmMYB195
GmMYB136
GmMYB175
GmMYB178
GmMYB194
FaMYB1
GmMYB170
GmMYB203
GmMYB177
GmMYB211
GmMYB106
GmMYB006
GmMYB141
HvMYB5
AtMYB3
AtMYB6
AtMYB8
Zm38
Zm42
AtMYB7
AtMYB32
GmMYB002
PhMYB4
AtMYB4
GmMYB005
GmMYB042
SbY1
ZmP1
ZmP2
GmMYB176
GmMYB190
GmMYB200
VvMYBF1
SlMYB12
AtMYB111
AtMYB11
AtMYB12
GmMYB174
GmMYB204
GmMYB197
GmMYB198
GmMYB098
GmMYB122
GmMYB116
GmMYB129
AtMYB20
AtMYB43
AtMYB85
AtMYB42
GmMYB097
GmMYB114
GmMYB101
GmMYB126
AtMYB99
Pp2
PtMYB1
AtMYB40
GmMYB124
GmMYB238
GmMYB239
GmMYB163
GmMYB232
GmMYB165
GmMYB209
AtMYB103
GmMYB185
GmMYB213
GmMYB184
GmMYB009
GmMYB187
AtMYB55
GmMYB094
GmMYB039
GmMYB023
GmMYB214
PtMYB8
AtMYB50
AtMYB61
HvMYB33
AtMYB86
GmMYB189
GmMYB186
GmMYB057
GmMYB162
GmMYB107
GmMYB121
GmMYB102
AtMYB26
AtMYB67
GmMYB065
GmMYB085
PtMYB4
GmMYB020
GmMYB188
GmMYB179
GmMYB202
AtMYB46
GmMYB015
GmMYB182
AtMYB83
EgMYB2
GmMYB118
GmMYB180
R2 R3
5'
1k 2k 3k 4k 5k 6k 7k 8k

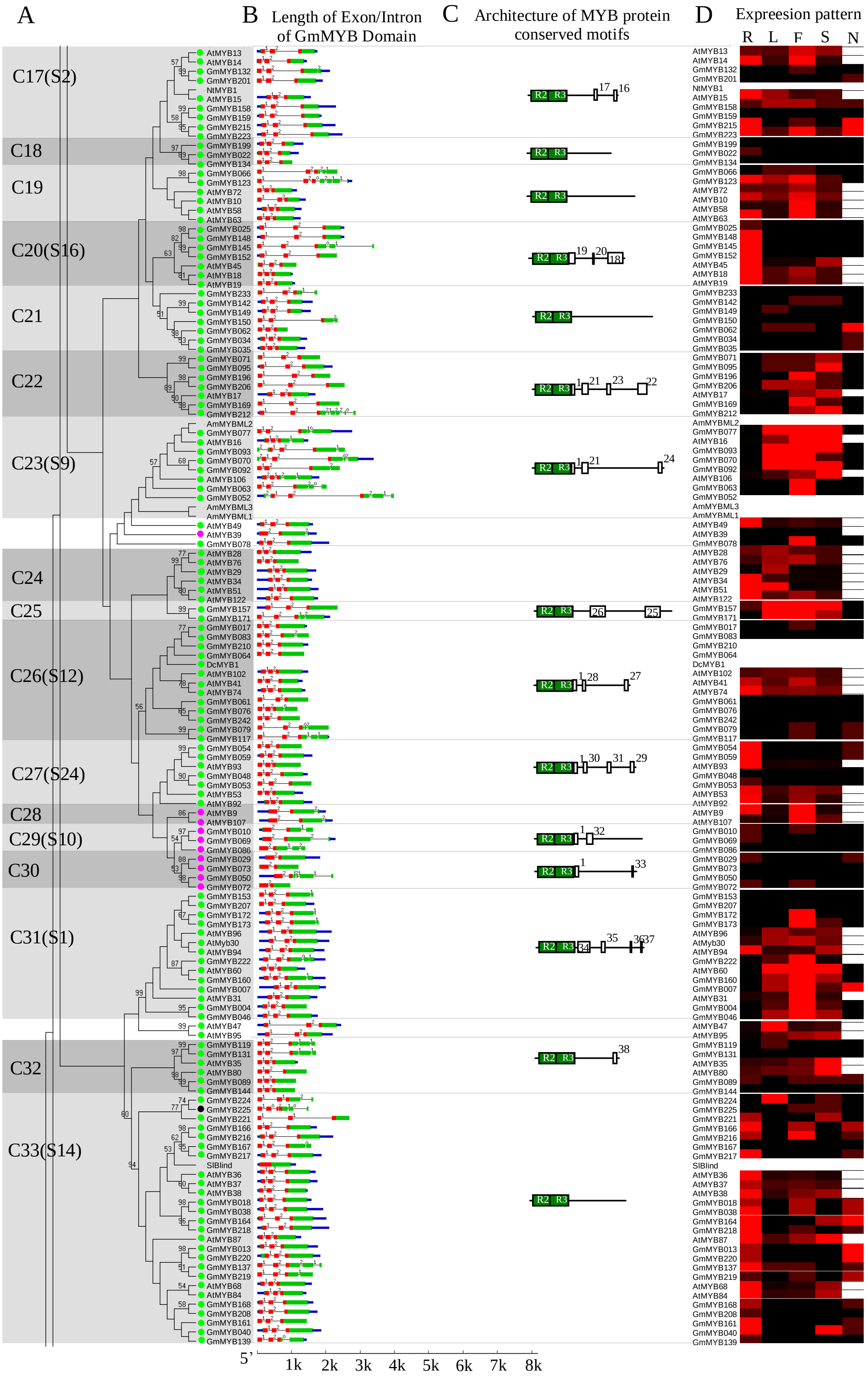

A
B Length of Exon/Intron of GmMYB Domain
C Architecture of MYB protein conserved motifs
D Expreesion pattern
R L F S N
C17(S2)
C18
C19
C20(S16)
C21
C22
C23(S9)
C24
C25
C26(S12)
C27(S24)
C28
C29(S10)
C30
C31(S1)
C32
C33(S14)
5'
1k 2k 3k 4k 5k 6k 7k 8k

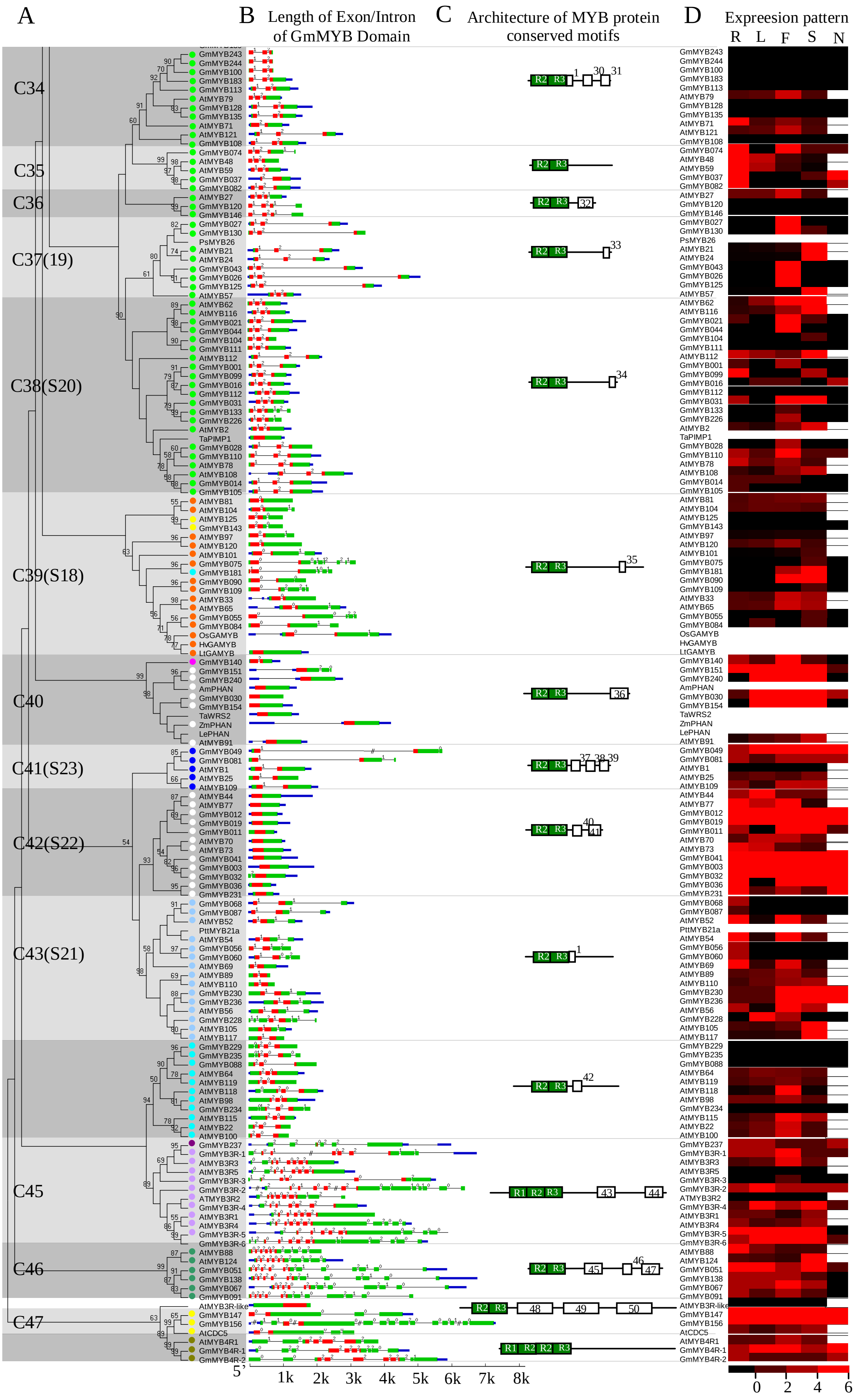
A
B Length of Exon/Intron of GmMYB Domain
C Architecture of MYB protein conserved motifs
D Expresion pattern
R L F S N
C34
C35
C36
C37(19)
C38(S20)
C39(S18)
C40
C41(S23)
C42(S22)
C43(S21)
C45
C46
C47
5'
1k 2k 3k 4k 5k 6k 7k 8k
0 2 4 6

Additional file 3. The complete version of Figure 2: Phylogenetic relationships and subgroup designations in MYB proteins from soybean (Gm), *Arabidopsis* (At) and other plants. (a) Neighbor-joining tree representing relationships among 252 MYB proteins from soybean and 132 MYB proteins from *Arabidopsis*, including five 3R-MYB proteins from *Arabidopsis* and six 3R-MYB proteins from soybean. The proteins are clustered into 47 subgroups, which are designated with a subgroup number (e.g., C1) and marked with different alternating tones of a gray background to facilitate subfamily identification with high predictive value. The numbers beside the branches represent bootstrap support values (>50%) from 1000 replications. Sixteen proteins did not fit well into clusters. Colored circles indicate the corresponding intron distribution patterns, as shown in Figure 3. (b) Structure of MYB genes in soybean and *Arabidopsis*. Exon(s) are indicated by green boxes, MYB domain(s) by red boxes, untranslated region(s) by blue boxes, and spaces between the colored boxes correspond to introns. The sizes of exons and introns can be estimated using the horizontal scale bar. (c) Architecture of conserved protein motifs in 47 subfamilies. The motifs on the right were detected using MEME and are graphically represented as white boxes drawn to scale for a representative plant MYB protein of each subfamily. (d) Expression patterns of MYB genes in soybean and *Arabidopsis* in different organs. R, root; L, leaf; F, flower; S, seed; N, legume-specific nodulation. In this expression pattern analysis, the highest values among the expression values of the four organs published in the AtGenExpress and SoySeq databases were selected.
