# Supplementary material for: Genome-wide analysis of the MYB transcription factor superfamily in soybean
Source: BMC Plant Biol. 2012 Jul 9;12:106. doi: 10.1186/1471-2229-12-106 (PMC3462118; doi:10.1186/1471-2229-12-106)
Supplement: Additional file 4 — MP phylogenetic tree of 387 MYB proteins in soybean,Aribidopsisand other plants. [file 1471-2229-12-106-S4.pdf]

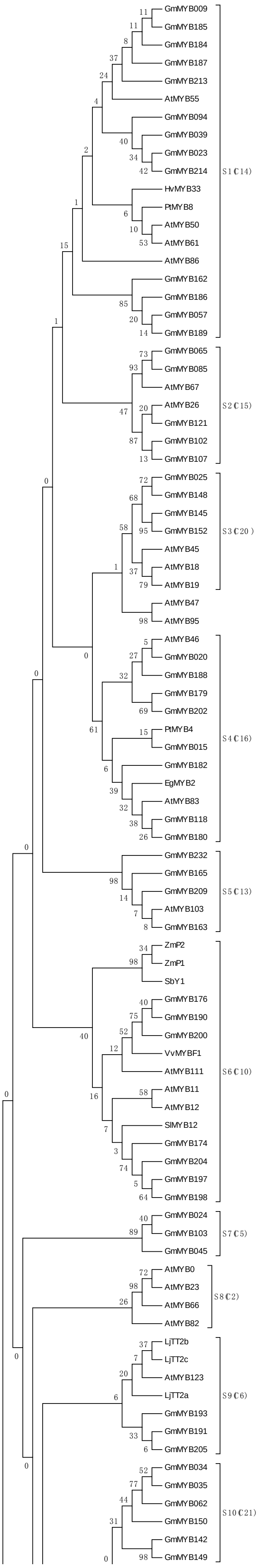

GmMYB009
GmMYB185
GmMYB184
GmMYB187
GmMYB213
AtMYB55
GmMYB094
GmMYB039
GmMYB023
GmMYB214
HvMYB33
PtMYB8
AtMYB50
AtMYB61
AtMYB86
GmMYB162
GmMYB186
GmMYB057
GmMYB189
S1 (14)
GmMYB065
GmMYB085
AtMYB67
AtMYB26
GmMYB121
GmMYB102
GmMYB107
S2 (15)
GmMYB025
GmMYB148
GmMYB145
GmMYB152
AtMYB45
AtMYB18
AtMYB19
S3 (20)
AtMYB47
AtMYB95
AtMYB46
GmMYB020
GmMYB188
GmMYB179
GmMYB202
PtMYB4
GmMYB015
GmMYB182
EgMYB2
AtMYB83
GmMYB118
GmMYB180
S4 (16)
GmMYB232
GmMYB165
GmMYB209
AtMYB103
GmMYB163
S5 (13)
ZmP2
ZmP1
SbY1
GmMYB176
GmMYB190
GmMYB200
VvMYBF1
AtMYB111
AtMYB11
AtMYB12
SlMYB12
GmMYB174
GmMYB204
GmMYB197
GmMYB198
S6 (10)
GmMYB024
GmMYB103
GmMYB045
S7 (5)
AtMYB0
AtMYB23
AtMYB66
AtMYB82
S8 (2)
LjTT2b
LjTT2c
AtMYB123
LjTT2a
GmMYB193
GmMYB191
GmMYB205
S9 (6)
GmMYB034
GmMYB035
GmMYB062
GmMYB150
GmMYB142
GmMYB149
S10 (21)

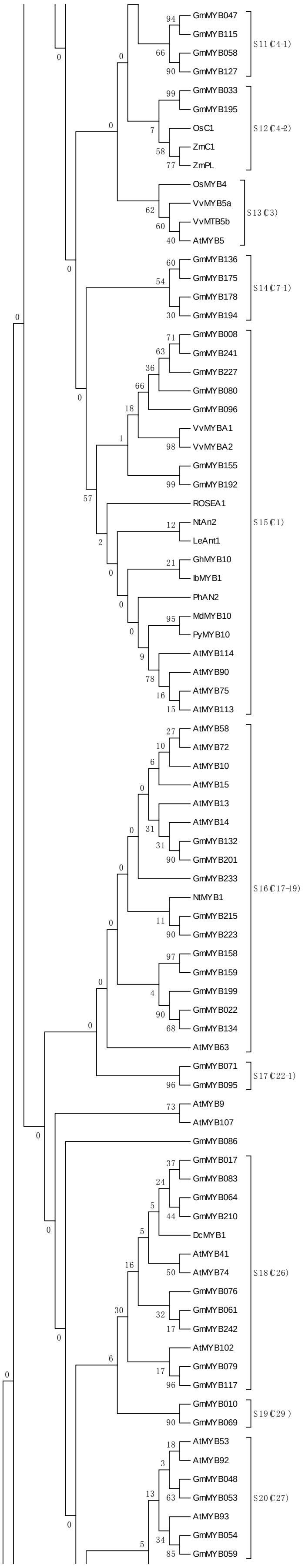
GmMYB047
GmMYB115
GmMYB058
GmMYB127
S11（C4-1）
GmMYB033
GmMYB195
OsC1
ZmC1
ZmPL
S12（C4-2）
OsMYB4
VvMYB5a
VvMTB5b
AtMYB5
S13（C3）
GmMYB136
GmMYB175
GmMYB178
GmMYB194
S14（C7-1）
GmMYB008
GmMYB241
GmMYB227
GmMYB080
GmMYB096
VvMYBA1
VvMYBA2
GmMYB155
GmMYB192
ROSEA1
NtAn2
LeAnt1
GhMYB10
IbMYB1
PhAN2
MdMYB10
PyMYB10
AtMYB114
AtMYB90
AtMYB75
AtMYB113
S15（C1）
AtMYB58
AtMYB72
AtMYB10
AtMYB15
AtMYB13
AtMYB14
GmMYB132
GmMYB201
GmMYB233
NtMYB1
GmMYB215
GmMYB223
GmMYB158
GmMYB159
GmMYB199
GmMYB022
GmMYB134
AtMYB63
S16（C17-19）
GmMYB071
GmMYB095
S17（C22-1）
AtMYB9
AtMYB107
GmMYB086
GmMYB017
GmMYB083
GmMYB064
GmMYB210
DcMYB1
AtMYB41
AtMYB74
GmMYB076
GmMYB061
GmMYB242
AtMYB102
GmMYB079
GmMYB117
S18（C26）
GmMYB010
GmMYB069
S19（C29）
AtMYB53
AtMYB92
GmMYB048
GmMYB053
AtMYB93
GmMYB054
GmMYB059
S20（C27）

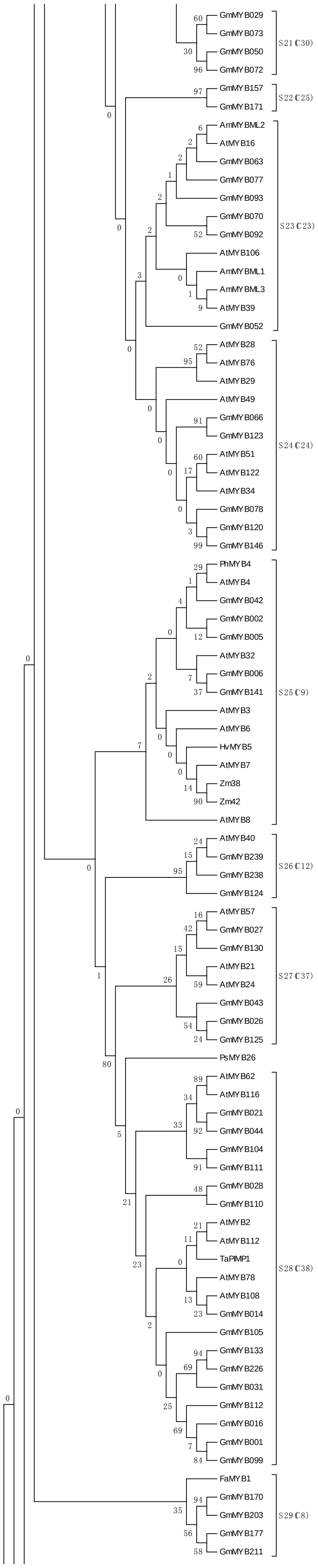
GmMYB029
GmMYB073
GmMYB050
GmMYB072
S21 (30)
GmMYB157
GmMYB171
S22 (25)
AmMYBML2
AtMYB16
GmMYB063
GmMYB077
GmMYB093
GmMYB070
GmMYB092
AtMYB106
AmMYBML1
AmMYBML3
AtMYB39
GmMYB052
S23 (23)
AtMYB28
AtMYB76
AtMYB29
AtMYB49
GmMYB066
GmMYB123
AtMYB51
AtMYB122
AtMYB34
GmMYB078
GmMYB120
GmMYB146
S24 (24)
PhMYB4
AtMYB4
GmMYB042
GmMYB002
GmMYB005
AtMYB32
GmMYB006
GmMYB141
AtMYB3
AtMYB6
HvMYB5
AtMYB7
Zm38
Zm42
AtMYB8
S25 (9)
AtMYB40
GmMYB239
GmMYB238
GmMYB124
S26 (12)
AtMYB57
GmMYB027
GmMYB130
AtMYB21
AtMYB24
GmMYB043
GmMYB026
GmMYB125
S27 (37)
PsMYB26
AtMYB62
AtMYB116
GmMYB021
GmMYB044
GmMYB104
GmMYB111
GmMYB028
GmMYB110
AtMYB2
AtMYB112
TaPIMP1
AtMYB78
AtMYB108
GmMYB014
GmMYB105
GmMYB133
GmMYB226
GmMYB031
GmMYB112
GmMYB016
GmMYB001
GmMYB099
S28 (38)
FaMYB1
GmMYB170
GmMYB203
GmMYB177
GmMYB211
S29 (8)

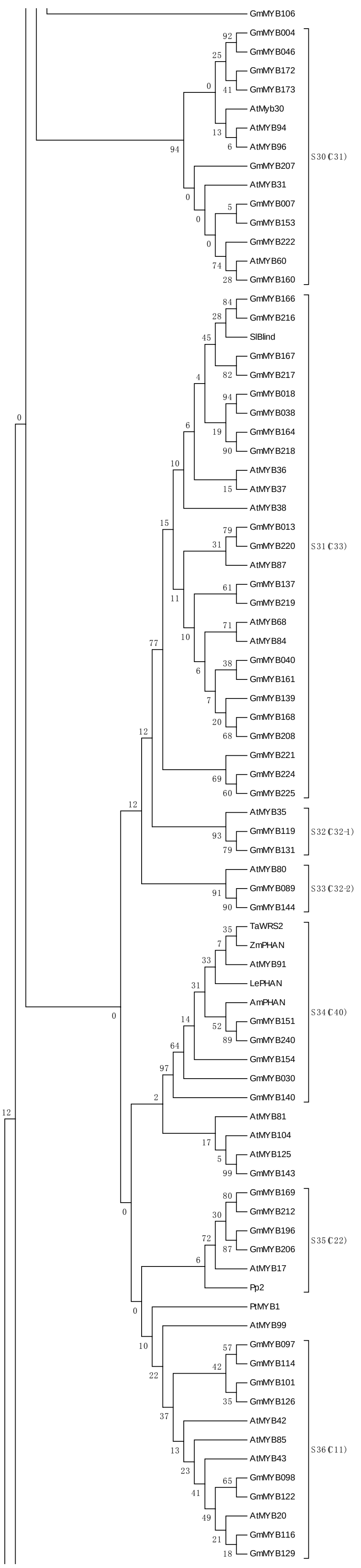
GmMYB106
GmMYB004
GmMYB046
GmMYB172
GmMYB173
AtMyb30
AtMYB94
AtMYB96
GmMYB207
AtMYB31
GmMYB007
GmMYB153
GmMYB222
AtMYB60
GmMYB160
S30 (31)
GmMYB166
GmMYB216
SlBlind
GmMYB167
GmMYB217
GmMYB018
GmMYB038
GmMYB164
GmMYB218
AtMYB36
AtMYB37
AtMYB38
GmMYB013
GmMYB220
AtMYB87
GmMYB137
GmMYB219
AtMYB68
AtMYB84
GmMYB040
GmMYB161
GmMYB139
GmMYB168
GmMYB208
GmMYB221
GmMYB224
GmMYB225
S31 (33)
AtMYB35
GmMYB119
GmMYB131
S32 (32-1)
AtMYB80
GmMYB089
GmMYB144
S33 (32-2)
TaWRS2
ZmPHAN
AtMYB91
LePHAN
AmPHAN
GmMYB151
GmMYB240
GmMYB154
GmMYB030
GmMYB140
S34 (40)
AtMYB81
AtMYB104
AtMYB125
GmMYB143
GmMYB169
GmMYB212
GmMYB196
GmMYB206
AtMYB17
Pp2
S35 (22)
PtMYB1
AtMYB99
GmMYB097
GmMYB114
GmMYB101
GmMYB126
AtMYB42
AtMYB85
AtMYB43
GmMYB098
GmMYB122
AtMYB20
GmMYB116
GmMYB129
S36 (11)

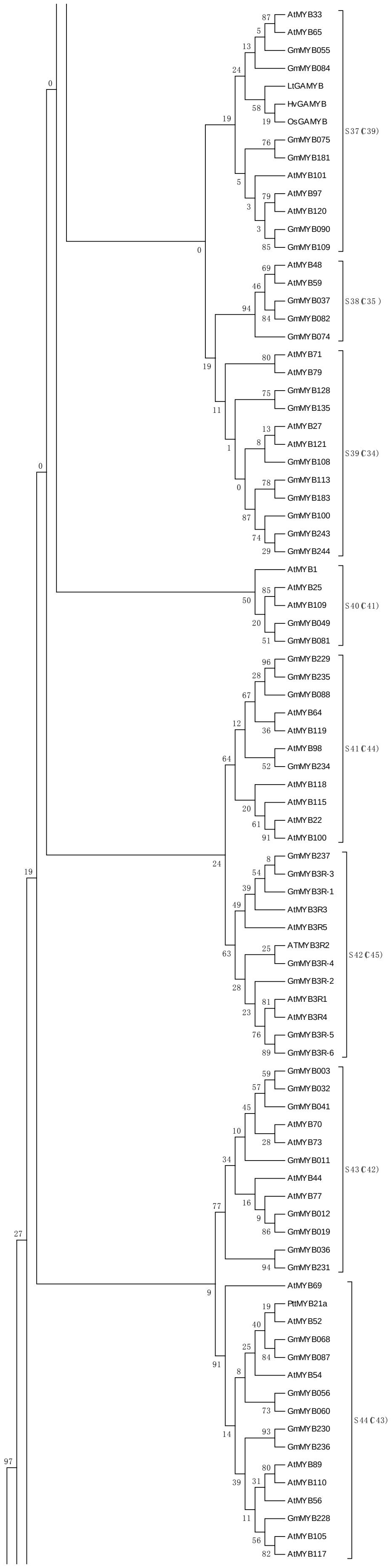
AtMYB33
AtMYB65
GmMYB055
GmMYB084
LtGAMYB
HvGAMYB
OsGAMYB
GmMYB075
GmMYB181
AtMYB101
AtMYB97
AtMYB120
GmMYB090
GmMYB109
S37(39)
AtMYB48
AtMYB59
GmMYB037
GmMYB082
GmMYB074
S38(35)
AtMYB71
AtMYB79
GmMYB128
GmMYB135
AtMYB27
AtMYB121
GmMYB108
GmMYB113
GmMYB183
GmMYB100
GmMYB243
GmMYB244
S39(34)
AtMYB1
AtMYB25
AtMYB109
GmMYB049
GmMYB081
S40(41)
GmMYB229
GmMYB235
GmMYB088
AtMYB64
AtMYB119
AtMYB98
GmMYB234
AtMYB118
AtMYB115
AtMYB22
AtMYB100
S41(44)
GmMYB237
GmMYB3R-3
GmMYB3R-1
AtMYB3R3
AtMYB3R5
ATMYB3R2
GmMYB3R-4
GmMYB3R-2
AtMYB3R1
AtMYB3R4
GmMYB3R-5
GmMYB3R-6
S42(45)
GmMYB003
GmMYB032
GmMYB041
AtMYB70
AtMYB73
GmMYB011
AtMYB44
AtMYB77
GmMYB012
GmMYB019
GmMYB036
GmMYB231
S43(42)
AtMYB69
PttMYB21a
AtMYB52
GmMYB068
GmMYB087
AtMYB54
GmMYB056
GmMYB060
GmMYB230
GmMYB236
AtMYB89
AtMYB110
AtMYB56
GmMYB228
AtMYB105
AtMYB117
S44(43)

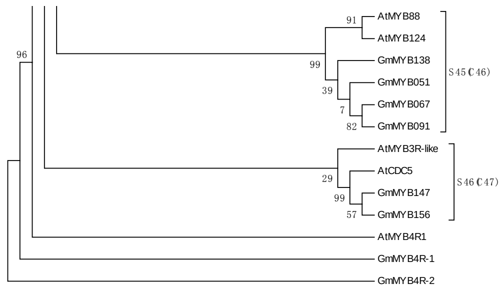

Additional file 4: MP phylogenetic tree of 387 MYB proteins in soybean and *Aribidopsis*. The unrooted tree was constructed by the max parsimony method based on the alignment of the corresponding MYB domains. Boot strapping values are indicated as percentages along the branches in MP analysis. The MYB proteins are clustered into 46 subgroups (designated as S1 to S46) and the corresponding subfamilies in NJ tree (Figure 2) were listed in round bracket, for reference.
