# Supplementary material for: Genome-wide analysis of the MYB transcription factor superfamily in soybean
Source: BMC Plant Biol. 2012 Jul 9;12:106. doi: 10.1186/1471-2229-12-106 (PMC3462118; doi:10.1186/1471-2229-12-106)
Supplement: Additional file 6 — NJ phylogenetic tree of 244 soybean R2R3-MYB proteins. [file 1471-2229-12-106-S6.pdf]

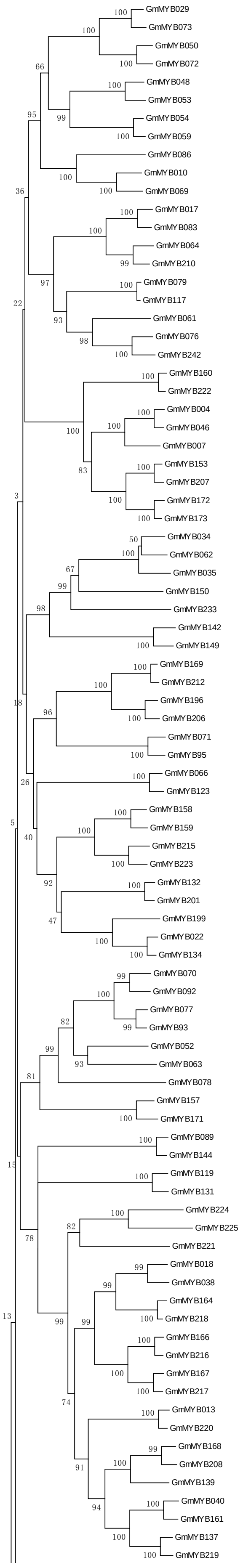
GmMYB029
GmMYB073
GmMYB050
GmMYB072
GmMYB048
GmMYB053
GmMYB054
GmMYB059
GmMYB086
GmMYB010
GmMYB069
GmMYB017
GmMYB083
GmMYB064
GmMYB210
GmMYB079
GmMYB117
GmMYB061
GmMYB076
GmMYB242
GmMYB160
GmMYB222
GmMYB004
GmMYB046
GmMYB007
GmMYB153
GmMYB207
GmMYB172
GmMYB173
GmMYB034
GmMYB062
GmMYB035
GmMYB150
GmMYB233
GmMYB142
GmMYB149
GmMYB169
GmMYB212
GmMYB196
GmMYB206
GmMYB071
GmMYB95
GmMYB066
GmMYB123
GmMYB158
GmMYB159
GmMYB215
GmMYB223
GmMYB132
GmMYB201
GmMYB199
GmMYB022
GmMYB134
GmMYB070
GmMYB092
GmMYB077
GmMYB93
GmMYB052
GmMYB063
GmMYB078
GmMYB157
GmMYB171
GmMYB089
GmMYB144
GmMYB119
GmMYB131
GmMYB224
GmMYB225
GmMYB221
GmMYB018
GmMYB038
GmMYB164
GmMYB218
GmMYB166
GmMYB216
GmMYB167
GmMYB217
GmMYB013
GmMYB220
GmMYB168
GmMYB208
GmMYB139
GmMYB040
GmMYB161
GmMYB137
GmMYB219

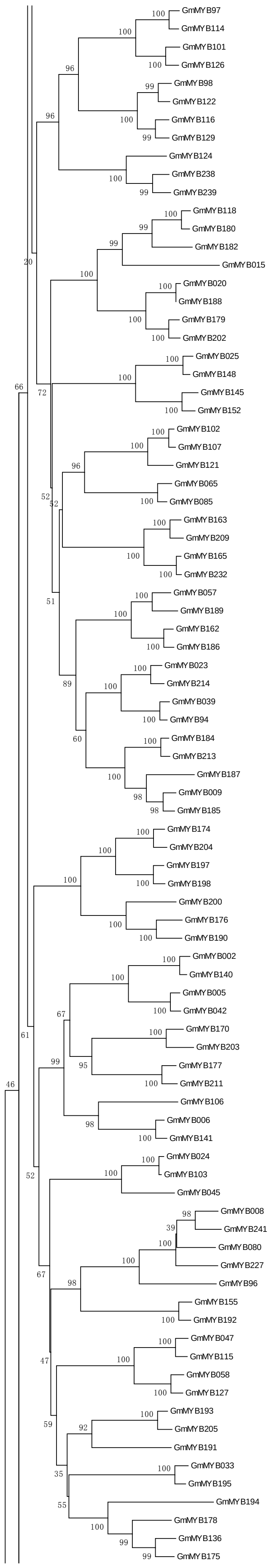

GmMYB97
GmMYB114
GmMYB101
GmMYB126
GmMYB98
GmMYB122
GmMYB116
GmMYB129
GmMYB124
GmMYB238
GmMYB239
GmMYB118
GmMYB180
GmMYB182
GmMYB015
GmMYB020
GmMYB188
GmMYB179
GmMYB202
GmMYB025
GmMYB148
GmMYB145
GmMYB152
GmMYB102
GmMYB107
GmMYB121
GmMYB065
GmMYB085
GmMYB163
GmMYB209
GmMYB165
GmMYB232
GmMYB057
GmMYB189
GmMYB162
GmMYB186
GmMYB023
GmMYB214
GmMYB039
GmMYB94
GmMYB184
GmMYB213
GmMYB187
GmMYB009
GmMYB185
GmMYB174
GmMYB204
GmMYB197
GmMYB198
GmMYB200
GmMYB176
GmMYB190
GmMYB002
GmMYB140
GmMYB005
GmMYB042
GmMYB170
GmMYB203
GmMYB177
GmMYB211
GmMYB106
GmMYB006
GmMYB141
GmMYB024
GmMYB103
GmMYB045
GmMYB008
GmMYB241
GmMYB080
GmMYB227
GmMYB96
GmMYB155
GmMYB192
GmMYB047
GmMYB115
GmMYB058
GmMYB127
GmMYB193
GmMYB205
GmMYB191
GmMYB033
GmMYB195
GmMYB194
GmMYB178
GmMYB136
GmMYB175

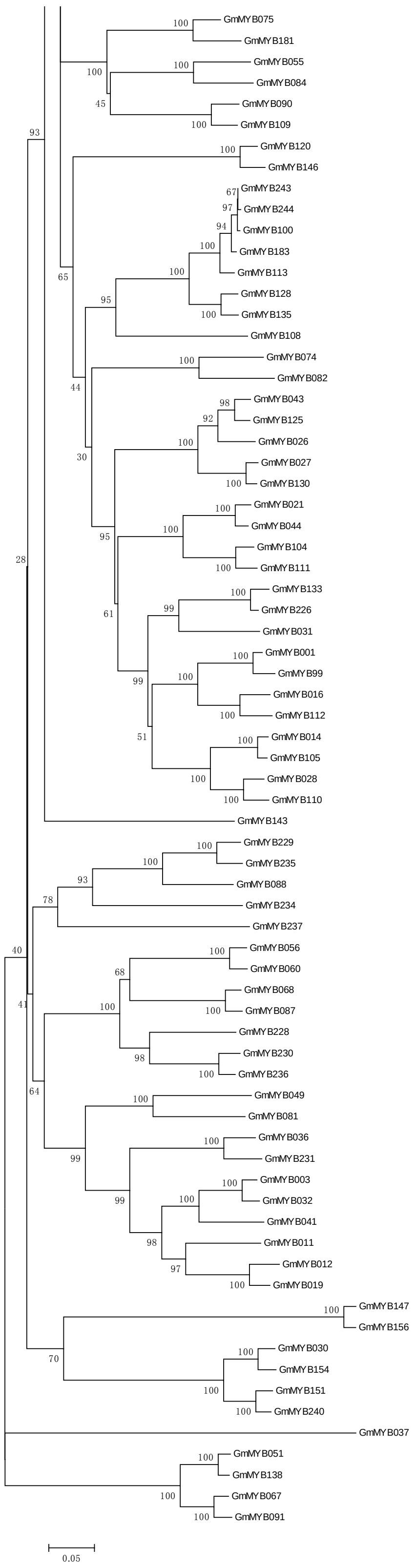

Additional file 6: NJ phylogenetic tree of 244 soybean R2R3-MYB proteins. The unrooted tree was constructed by the NJ method based on the alignment of the 244 soybean MYB domains. Bootstrapping values are indicated as percentages along the branches in NJ analysis.
